# Time-dependent ROC curve analysis in medical research: current methods and applications

**DOI:** 10.1186/s12874-017-0332-6

**Published:** 2017-04-07

**Authors:** Adina Najwa Kamarudin, Trevor Cox, Ruwanthi Kolamunnage-Dona

**Affiliations:** Department of Biostatistics, University of Liverpool, Liverpool, L69 3GL UK

**Keywords:** ROC curve, Time-dependent AUC, Biomarker evaluation, Event-time, Longitudinal data, Software

## Abstract

**Background:**

ROC (receiver operating characteristic) curve analysis is well established for assessing how well a marker is capable of discriminating between individuals who experience disease onset and individuals who do not. The classical (standard) approach of ROC curve analysis considers event (disease) status and marker value for an individual as fixed over time, however in practice, both the disease status and marker value change over time. Individuals who are disease-free earlier may develop the disease later due to longer study follow-up, and also their marker value may change from baseline during follow-up. Thus, an ROC curve as a function of time is more appropriate. However, many researchers still use the standard ROC curve approach to determine the marker capability ignoring the time dependency of the disease status or the marker.

**Methods:**

We comprehensively review currently proposed methodologies of time-dependent ROC curves which use single or longitudinal marker measurements, aiming to provide clarity in each methodology, identify software tools to carry out such analysis in practice and illustrate several applications of the methodology. We have also extended some methods to incorporate a longitudinal marker and illustrated the methodologies using a sequential dataset from the Mayo Clinic trial in primary biliary cirrhosis (PBC) of the liver.

**Results:**

From our methodological review, we have identified 18 estimation methods of time-dependent ROC curve analyses for censored event times and three other methods can only deal with non-censored event times. Despite the considerable numbers of estimation methods, applications of the methodology in clinical studies are still lacking.

**Conclusions:**

The value of time-dependent ROC curve methods has been re-established. We have illustrated the methods in practice using currently available software and made some recommendations for future research.

**Electronic supplementary material:**

The online version of this article (doi:10.1186/s12874-017-0332-6) contains supplementary material, which is available to authorized users.

## Background

In a screening process, an appropriate marker is used to provide information on the individual risk of disease onset. Information and signalling of future disease identification may be given by a single continuous measurement marker or a score. A single measurement could be any clinical measure such as cell percentage in the synthesis phase to detect breast cancer [1], CD4 cell counts to detect AIDS [2] or HIV-1 RNA to detect HIV [3]. A score from a regression of potential factors or some other model to detect disease can also be used as a marker. Chambless and Diao [4] used the score from a logistic regression model, including several traditional and newer risk factors, to detect Coronary Heart Disease (CHD). Lambert and Chevret [5] used the prognostic score of four covariates (age, platelet count, prothrombin time, and serum alpha-fetoprotein level) to predict compensated cirrhosis patients’ survival and also used a score of three baseline characteristics (age, white blood cell and performance status) to predict event-free survival (EFS) in acute leukaemia patients. Moreover, some studies used a published score as a marker in which the score considers the most important mortality predictors of a certain disease. For example, the Framingham risk score is used for cardiovascular patients [6] and the Karnofsky score is used for lung cancer patients [7].

The decision from a diagnostic test is often based on whether the marker value exceeds a threshold value, in which case the diagnosis for the individual is “diseased” and “non-diseased” otherwise. There is a possibility that the diagnostic test gives a positive result for a non-diseased individual or a negative result for a diseased individual. The sensitivity is defined as the probability of a diseased individual being predicted as having the disease (true-positive) and the specificity as the probability of a non-diseased individual being predicted as not having the disease (true-negative). These probabilities change as the threshold value for the marker changes and the value or range of threshold values chosen depends on the trade-off that is acceptable between failing to detect disease and falsely identifying disease with the test [8]. In relation to this, the receiver operating characteristic (ROC) curve is a tool that simply describes the range of trade-offs achieved by a diagnostic test. ROC curve analysis is extensively used in biomedical studies for evaluating the diagnostic accuracy of a continuous marker. It is a graphical display which plots sensitivity estimates (probability of a true positive) against one minus specificity (probability of a false positive) of a marker for all possible threshold values. The performance of a marker is evaluated by the area under the ROC curve (AUC) in which a higher AUC value indicates a better marker performance. The AUC is also equal to the probability of a diseased individual having a higher marker value than a healthy individual [8]. It is usually assumed that a higher marker value is more indicative of disease [8, 9] and we assume this for the rest of this article.

Recent research has incorporated time dependency in the sensitivity and specificity in disease (event)-time data for individuals instead of using the standard ROC curve approach. Such methods are proven more effective; however, these methods are still under-used in medical research. Once the time-dependent setting has been applied, the disease status is observed at each time point which yields different values of sensitivity and specificity throughout the study.

Let *T*_*i*_ denote the time of disease onset and *X*_*i*_ is a marker value (usually the value at baseline) for individual *i*, (*i* = 1, …, *n*). Define the observed event time, *Z*_*i*_ = *min*(*T*_*i*_, *C*_*i*_), where *C*_*i*_ is a censoring time, and let *δ*_*i*_ be the censoring indicator taking value 1 if an event (disease) occurs and 0 otherwise. Let *D*_*i*_(*t*) be the disease status at time *t*, taking values 1 or 0. Hereafter, we will refer to *X* as a “marker”, but *X* may also denote a risk score computed from a regression or some other model, or a published score. For a given threshold *c*, the time-dependent sensitivity and specificity can defined respectively by

$$ \begin{array}{l} Se\left( c, t\right)= P\left({X}_i> c\Big|{D}_i(t)=1\right)\\ {} Sp\left( c, t\right)= P\left({X}_i\le c\Big|{D}_i(t)=0\right).\end{array} $$

Using the above definitions, we can define the corresponding ROC curve for any time *t* as *ROC(t)* which plots *Se*(*c,t*) against 1-*Sp*(*c,t*) for thresholds *c* and time-dependent AUC is defined by

$$ A U C(t)={\displaystyle \underset{-\infty }{\overset{\infty }{\int }}} S e\left( c, t\right) d\left[1- Sp\left( c, t\right)\right] $$

with $$ \left[1- Sp\left( c, t\right)\right]=\frac{\partial \left[1- Sp\left( c, t\right)\right]}{\partial c} dc. $$

The AUC is equal to the probability that the diagnostic test results from a randomly selected pair of diseased and non-diseased individuals are correctly ordered [10, 11].

**Fig. 1:**
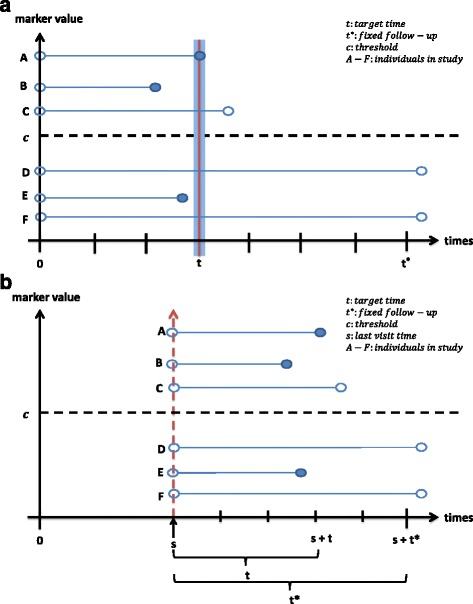
**a** Illustration for cases and controls of C/D, I/D and I/S (baseline) definitions. C/D: A, B and E are cases and C, D and F are controls; I/D: Only A is the case and C, D and F are controls; I/S: Only A is the case and D and F are controls. **b** Illustration for cases and controls of I/S (longitudinal) definitions. Only A is the case and D and F are the controls

Heagerty and Zheng [12] proposed three different definitions for estimating the above time-dependent sensitivity and specificity for censored event-times, namely (1) cumulative/dynamic (C/D), (2) incident/dynamic (I/D) and (3) incident/static (I/S) and these are explained by referring to the illustrations in Fig. 1(a) and (b) below. Figure 1(a) and (b) illustrate the cases and controls that contribute to the three definitions of sensitivity and specificity (C/D and I/D with the baseline marker, and I/S with both the baseline and longitudinal markers), with closed circles indicate individuals who had an event, open circles indicate individuals who had censored event-times.

### *Cumulative* sensitivity and *dynamic* specificity (C/D)

At each time point *t*, each individual is classified as a case or control. A case is defined as any individual experiencing the event between baseline *t* = 0 and time *t* (individual A, B or E in Fig. 1a) and a control as an individual remaining event-free at time *t* (individual C, D or E in Fig. 1a). The cases and controls are changing over time and each individual may play the role of control at the earlier time (when the event time is greater than the target time, i.e. *T*_*i*_ > *t*) but then contributes as a case for later times (when the event time is less than or equal to the target time, i.e.*T*_*i*_ ≤ *t*).

The cumulative sensitivity is the probability that an individual has a marker value greater than *c* among the individuals who experienced the event **before time** ***t*** (individual A or B in Fig. 1a), and the dynamic specificity is the probability that an individual has a marker value less than or equal to *c* among those event-free individuals **beyond time** ***t*** (individual D or F in Fig. 1a). Thus the sensitivity and specificity at time *t* and the resulting *AUC*(*t*) can be defined as

$$ \begin{array}{l} S{e}^C\left( c, t\right)= P\left({X}_i> c\Big|{T}_i\le t\right)\\ {} S{p}^D\left( c, t\right)= P\left({X}_i\le c\Big|{T}_i> t\right)\\ {} AU{C}^{C, D}(t)= P\left({X}_i>{X}_j\Big|{T}_i\le t,{T}_j> t\right), i\ne j.\end{array} $$

It is more appropriate to apply the C/D definitions when there is a specific time of interest that is used to discriminate between individuals experiencing the event and those event-free prior to the specific time. This type of discrimination has more clinical relevance than the other definitions (I/D and I/S) and hence C/D definition has commonly been used by clinical applications [5, 13]. However, since some individuals may contribute as controls at an earlier time and then contribute as cases later, this definition uses redundant information in separating cases and controls [5].

### *Incident* sensitivity and *dynamic* specificity (I/D)

A case for I/D definition is defined as an individual with an event at time *t* (individual A in Fig. 1a) while the control is an event-free individual at time *t*. (individual C, D or F in Fig. 1a). In this definition, there are individuals neither a control nor case (when the event time is less than the target time, i.e. *T*_*i*_ < *t*, individual B or E in Fig. 1a). Each individual who had an event may play the role of control at the earlier time (when the event time is greater than target time, i.e. *T*_*i*_ > *t*) but then contributes as a case at the later incident time (when the event time is the same as the target time, i.e.*T*_*i*_ = *t*).

The incident sensitivity is the probability that an individual has a marker value greater than *c* among the individuals who experience the event **at time** ***t*** (individual A in Fig. 1a) and the dynamic specificity is the probability that an individual has a marker value less than or equal to *c* among the individuals that remain event-free **at time** ***t*** (individual D or F in Fig. 1a). The sensitivity, specificity and resulting *AUC*(*t*) are defined as

$$ \begin{array}{l} S{e}^I= P\left({X}_i> c\Big|{T}_i= t\right)\\ {} S{p}^D= P\left({X}_i\le c\Big|{T}_i> t\right)\\ {} AU{C}^{I, D}(t)= P\left({X}_i>{X}_j\Big|{T}_i= t,{T}_j> t\right), i\ne j.\end{array} $$

The I/D terminology is more appropriate when the exact event time is known and we want to discriminate between individuals experiencing the event and those event-free at a given event-time, i.e. *T*_*i*_ = *t*. The incident sensitivity and dynamic specificity are defined by dichotomizing the riskset at time *t* into cases and controls and this is a natural companion to hazard models [12]. In addition, these definitions allow an extension to time-dependent covariates and also allow time-averaged summaries that directly relate to a familiar concordance measure c-statistic [12]. This is a special advantage of the I/D definition, since in many applications no a prior time *t* is identified, thus a global accuracy summary is usually desired. The concordance summary is a weighted average of the area under the time-dependent ROC curve and it is defined by Heagerty and Zheng [12] as

$$ {C}^{\tau}={\displaystyle {\int}_0^{\tau}} AU{C}^{I, D}(t){w}^{\tau}(t) d t $$

where ${w}^{\tau}(t)=2 f(t) S(t)/{W}^{\tau},{W}^{\tau}={\displaystyle {\int}_0^{\tau}}2 f(t) S(t) d t=1-{S}^2\left(\tau \right).$ The *C* ^*τ*^ has slightly different interpretation from the original concordance and it is the probability that the predictions for a random pair of individuals are concordant with their outcome, given that the smaller event time occurs in (0, *τ*).

### *Incident* sensitivity and *static* specificity (I/S)

A case for I/S definition is defined as an individual with an event at time *t* (individual A in Fig. 1a, while the control is an event-free individual through a fixed follow-up period, (0, t*) (individual D or F in Fig. 1a). This incident sensitivity and static specificity is usually used when a researcher attempts to distinguish between individuals who have an event at time *t* and those ‘long term survivors’ who are event-free after a suitably long follow-up time, characterized by *T*_*i*_ ≥ t*. The rational of using the fixed follow-up is because the end point t* is pre-specified and it is considered a long enough time to observe the event. For example, t* = 2 years is typically used in screening for breast cancer since it is assumed that the individual was free from subclinical disease if the clinical disease does not emerge by two years after screening [6]. The sensitivity and specificity can be defined by

$$ \begin{array}{l} S{e}^I\left( c, t\right)= P\left({X}_i> c\Big|{T}_i= t\right)\\ {} S{p}^S\left( c,{t}^{*}\right)= P\left({X}_i\le c\Big|{T}_i>{t}^{*}\right).\end{array} $$

As illustrated in Fig. 1a, the controls are static and do not change (individuals D and F), and each individual only contributes once as a case or as event-free individual within the fixed follow-up (0, t*).

The I/S definition can also be used in studies in which individuals are followed up for a fixed time period with repeated biomarker measurements. However, not all longitudinally measured marker values of the individual will be used, but only a marker value at a particular visit time *s* instead of using the baseline marker value [6, 14]. Since some studies may not have a regular visit time schedule, the visit times may differ for each individual. Thus, the time lag between the visit time and the time of disease onset, *T*_*i*_ − *s*, which is commonly termed by the “time prior to event”, is the main interest. The I/S definition with a longitudinally measured marker is illustrated in Fig. 1b, assuming that a marker value is measured at visit time *s*. The sensitivity and specificity are defined based on a time lag *t* = *T*_*i*_ − *s*. The incident sensitivity is the probability of test positive with the marker at *t* time units prior to the event for an individual that has an event at *T*_*i*_ (individual A in Fig. 1b). The static specificity is the probability that an individual is remained event free by t* time units after the marker is measured (individual D or F in Fig. 1b). We use *Y* instead of *X* to represent the longitudinal marker measurements in order to distinguish with the baseline marker value. Let *Y*_*ik*_ be the biomarker value obtained from individual *i* at *s*_*ik*_; *i* = 1, …, *n*; *k* = 1, …, *K*_*i*_ where *s*_*ik*_ is the marker measurement time of individual *i* at the *k* ^*th*^ visit time. The sensitivity and specificity can be defined by:

$$ \begin{array}{l} S{e}^I\left( c, t\right)= P\left({Y}_{i k}> c\Big|{T}_i-{s}_{i k}= t\right)\\ {} S{p}^S\left( c,{t}^{*}\right)= P\left({Y}_{i k}\le c\Big|{T}_i-{s}_{i k}>{t}^{*}\right).\end{array} $$

The above definitions facilitate the use of standard regression approaches for characterizing sensitivity and specificity because the time prior to event *T*_*i*_ − *s*_*ik*_ can simply be used as a covariate.

Blanche et al. [13] have reviewed methodologies of time-dependent ROC curve analysis under the C/D definition only; however, in this article, we have undertaken a comprehensive review of the current estimation methods under each definition and also identify additional methods, aiming to provide clarity for each methodology. We illustrate how each method is implemented on a time-varying disease status or over a time course of a longitudinal marker using a sequential dataset from Mayo Clinic trial in primary biliary cirrhosis (PBC) of the liver. We identify the software that implements current methods in practice and the need for further methodologies.

### Benefits of time-dependent ROC curve analysis

In the standard ROC curve analysis, the individual’s disease status is defined once a marker value is measured and it is assumed to be fixed for the whole study period. The study period is usually take a long follow-up and during this, the individual without disease earlier may have the disease. In contrast, the disease status of an individual is observed and updated at each time point in time-dependent ROC curve analysis. With additional information of time of disease onset for each individual, a ROC curve can be constructed at several time points and the marker’s predictive ability can be compared. Thus, time-dependent ROC curve is an efficient tool in measuring the performance of a candidate marker given the true disease status of individuals at certain time points. In general, a baseline marker value is used for computing the predictive ability but it can become weaker as the target time gets further from the baseline.

In longitudinal studies, the marker is measured several times within a fixed follow-up. If a marker measurement has ability to signify a pending change in the clinical status of a patient, then a time-dependent ROC curve on a time-varying marker can be used to guide key medical decisions.

### Challenges of time-dependent ROC curve analysis

The most common problem is censoring, in which some individuals may be lost during the follow-up period. If the censored individuals are ignored, the estimation of the sensitivity and specificity may be biased as the information from the individual before censoring may contribute to the estimation. In a time-dependent ROC curve analysis, the sensitivity and specificity are defined at each time point, where not all individuals are equally informative, and their contributions differing according to the aims and definitions used. A longitudinal biomarker brings an additional challenge to take account of the marker measurements at a number of visits for each individual. In the I/S definition, not all marker values are used but only the most recent, which is assumed more reliable for predicting the disease status [6, 14]. Other time-dependent ROC curve approaches currently proposed for a longitudinal marker either assume non-censored event-times or ignore censored individual records.

## Methods

We have used MEDLINE (Ovid), Scopus and the internet to search for relevant papers for our review. We restricted our search to English language published papers between years 1995 to August 2016 to ensure all methodology papers of time-dependent ROC curves analysis were included. A total of 332 papers were found and 24 of these discussed time-dependent ROC curve methodology. The remaining 308 papers included only an application of standard or time-dependent ROC curves. For each methodology paper, the following details were extracted: definition of sensitivity and specificity (whether C/D, I/D, I/S or other), estimation method, type of estimation (non-parametric, semi-parametric or parametric), limitations and availability of software. Only 16 methodology papers are within the scope of this review, and out of the 16 methodology papers, 10 (63%) discussed methodologies along the lines of the C/D definition. Three papers (19%) proposed methodologies based on the I/D definition, only one paper (6%) proposed methodology based on the I/S and another two papers (12%) proposed other methodologies for longitudinal marker measurements. Full details of the review are available as Additional file 1.

Table 1 summarised the estimation methods for each definition with their respective advantage and disadvantage and software tools. We discuss the methodologies proposed under each definition in detail in the subsequent sections.

**Table 1:** Summary of current methods for each definition

Definition and marker time	Sensitivity and specificity	Estimation method and R software (when available)	Pros/Cons	
C/D *t* = 0	$$ \begin{array}{l} S{e}^C\left( c, t\right)= P\left({X}_i> c\Big|{T}_i\le t\right)\\ {} S{p}^D\left( c, t\right)= P\left({X}_i\le c\Big|{T}_i> t\right)\end{array} $$	CD1 survivalROC	Pro: Easy Cons: a) Produce non-monotone sensitivity and specificity b) Not robust to marker-dependent censoring	Pro: Clinically relevant since many clinical experiments aim to discriminate individuals with disease prior to specific time and healthy individual beyond that time Con: Use redundant information in separating cases and controls
		CD2 survivalROC	Pros: a) Produce monotone sensitivity and specificity b) Allow censoring to depend on marker Con: Does involve smoothing parameter	
		CD3 Programme code	Pro: Does not involve any smoothing parameter Cons: a) Does involve recursive computation b) Produce non-monotone specificity	
		CD4 survAUC	Pros: a) Produce monotone sensitivity and specificity if the score is produced from a survival model b) Allow censoring to depend on marker Con: Not invariant to an increasing transformation of the marker	
		CD5 timeROC	Pros: a) Produce monotone sensitivity and specificity b) More robust than CD1 and CD3 Con: Does not robust to marker dependent censoring	
		CD6 timeROC	Pros: a) Produce monotone sensitivity and specificity b) Robust to marker-dependent censoring c) More less biased than CD2 when censoring strongly depends on marker	
		CD8, VL Cox survival	Pro: Straightforward to implement	
		VL Aalen timereg		
		VL KM prodlim		
C/D A longitudinal time point	$$ \begin{array}{l} S{e}^C\left( c, t\right)= P\left({Y}_{i k}> c\Big|{T}_i-{s}_{i k}\le t\right)\\ {} S{p}^D\left( c,{t}^{*}\right)= P\left({Y}_{i k}\le c\Big|{T}_i-{s}_{i k}> t\right)\end{array} $$	AD4 (ECD2)	Pros: a) Produce monotone sensitivity and specificity b) Allow censoring to depend on marker Con: Does involve smoothing parameter	Pro: Use the most recent marker value prior to prediction time Con: Just use a marker value at a particular time instead of using all serial of marker value
I/D *t* = 0	$$ \begin{array}{l} S{e}^I\left( c, t\right)= P\left({X}_i> c\Big|{T}_i= t\right)\\ {} S{p}^D\left( c, t\right)= P\left({X}_i\le c\Big|{T}_i> t\right)\end{array} $$	ID1 risksetROC	Pros: Produce consistent sensitivity and specificity if the control set is unbiased	Pro: Allow time-averaged summaries that directly relate to a familiar concordance measures such as Kendall’s tau or c-index Con: Require an exact time of interest which often just a few individual has an event at a particular point
		ID2	Pro: Potentially more robust than ID1 Con: Computationally intensive	
		ID3 Programme code	Pros: a) Easier especially when involve a large number of marker b) Understandable since it is a “regression-type” model	
I/S *t* = 0	$$ \begin{array}{l} S{e}^I\left( c, t\right)= P\left({Y}_i> c\Big|{T}_i= t\right)\\ {} S{p}^S\left( c,{t}^{*}\right)= P\left({Y}_i\le c\Big|{T}_i>{t}^{*}\right)\end{array} $$	None	Pro: Allow separation of long-term survivors from healthy individual within a fixed follow-up Con: Require an exact time of interest which often just a few individual has an event at a particular point	
I/S A longitudinal time point	$$ \begin{array}{l} S{e}^I\left( c, t\right)= P\left({Y}_{i k}> c\Big|{T}_i-{s}_{i k}= t\right)\\ {} S{p}^S\left( c,{t}^{*}\right)= P\left({Y}_{i k}\le c\Big|{T}_i-{s}_{i k}>{t}^{*}\right)\end{array} $$	IS1	Pro: Provides unbiased estimates of model parameters of sensitivity and specificity Con: Computationally intensive since involve spline functions	Pro: Use the most recent marker value prior to prediction time Con: Just use a most recent of marker value instead of all marker values
		IS2	Pro: Use the most recent marker value prior to prediction time Con: not a natural companion to hazard models	
Other All longitudinal time points	*ROC*(*t*, *p*) = *S*[*a*_0_(*T*_*ik*_) + *a*_1_(*T*_*ik*_)*S* ^− 1^(*p*)]	AD1 Programme code	Pro: Use all marker value along visit times in the estimation of ROC curve Con: Do not incorporate censored outcomes	

### Naïve estimator of time-dependent ROC curve analysis

Many studies have used an empirical estimator as a basis for comparison with other estimation methods. This estimator only considers observed events and, the sensitivity and specificity are calculated by the observed proportions of true-positives and true-negatives respectively.

If a dataset does not have any censored events (that is, if all individuals have either experienced the event or remained event-free over the study follow-up and not left the study), the sensitivity at time *t* is estimated as the proportion of the individuals with marker value greater than threshold *c*, (i.e. *X*_*i*_ > *c*) among individuals experiencing the event before *t*. The specificity at time *t* is given by the proportion of the individuals with marker value less than or equal to *c*, (i. e. *X*_*i*_ ≤ *c*) among event-free individuals beyond time *t*. When there are censored event-times, the above estimators are computed by removing all the censored individuals before time point *t*. The sensitivity and specificity and the resulting *AUC*(*t*) can be estimated as follows

$$ \begin{array}{l}\widehat{Se}\left( c, t\right)=\frac{{\displaystyle {\sum}_{i=1}^n}{\delta}_i I\left({X}_i> c,{Z}_i\le t\right)}{{\displaystyle {\sum}_{i=1}^n}{\delta}_i I\left({Z}_i\le t\right)}\\ {}\widehat{Sp}\left( c, t\right)=\frac{{\displaystyle {\sum}_{i=1}^n} I\left({X}_i\le c,{Z}_i> t\right)}{{\displaystyle {\sum}_{i=1}^n} I\left({Z}_i> t\right)}\\ {}\widehat{AUC}(t)=\frac{{\displaystyle {\sum}_{i=1}^n}{\displaystyle {\sum}_{j=1}^n}{\delta}_i I\left({Z}_i\le t,{Z}_j> t\right) I\left({X}_i>{X}_j\right)}{{\displaystyle {\sum}_{i=1}^n}{\delta}_i I\left({Z}_i\le t\right){\displaystyle {\sum}_{j=1}^n} I\left({Z}_j> t\right)}\end{array} $$

where *i* and *j* are the indexes of two independent individuals, and *I*(.) is an indicator function. However, this estimation is often biased as it ignores the censoring distribution. The specificity estimate is consistent if censoring is independent of *X*_*i*_ and *T*_*i*_, while the sensitivity and AUC estimates may be biased since *T*_*i*_ will usually depends on *X*_*i*_ [13].

### *Cumulative* sensitivity and *dynamic* specificity (C/D)

Ten estimation methods have been proposed under C/D definition, and these are discussed in CD1 – CD8 below. CD8 describes three estimation methods.

#### (CD1) Kaplan-Meier estimator of Heagerty et al. [1]

Heagerty et al. [1] used the Kaplan-Meier estimator of the survival function [15] to estimate the time-dependent sensitivity and specificity. Using Bayes’ Theorem, the two quantities are defined by

$$ \widehat{S e}\left( c, t\right)=\frac{\left\{1-\widehat{S}\left( t\Big|{X}_i> c\right)\right\}\ \left(1-{\widehat{F}}_X(c)\right)}{1-\widehat{S}(t)},\widehat{S p}\left( c, t\right)=\frac{\widehat{S}\left( t\Big|{X}_i\le c\right){\widehat{F}}_X(c)}{\widehat{S}(t)} $$

where *Ŝ*(*t*) is the estimated survival function, *Ŝ*(*t*|*X*_*i*_ > *c*) is the estimated conditional survival function for the subset defined by *X* > *c* and ${\widehat{F}}_X(c)$ is the empirical distribution function of the marker, *X*.

However, this estimator yields non-monotone sensitivity and specificity, and not bounded in [0, 1]. This problem is illustrated by the authors using a hypothetical dataset, and is due to the quadrant probability estimator $\widehat{P}\left({X}_i> c,{T}_i> t\right)=\widehat{S}\left( t\Big|{X}_i> c\right)\left(1-{\widehat{F}}_X(c)\right)$ not necessarily producing a valid bivariate distribution as the redistribution to the right of the probability mass associated with censored observations will change as the conditioning set (*X* > *c*) changes. Another problem is that it is not robust to marker-dependent censoring since the conditional Kaplan-Meier estimator, *Ŝ*(*t*|*X*_*i*_ > *c*), assumes the censoring process does not depend on the marker.

#### (CD2) nearest neighbour estimator of Heagerty et al. [1]

The problems of the CD1 estimators motivated Heagerty et al. [1] to develop an alternative approach based on a bivariate survival function. This improved methodology uses the nearest neighbour estimator of the bivariate distribution of (*X*, *T*), introduced by Akritas [16]. As mentioned earlier, CD1 is not robust to marker-dependent censoring; however, censoring often depends on the marker. Thus, the independence of time-to-event and censoring time cannot be assumed and they are more likely independent conditionally on the marker. In this model-based approach, the probability of each individual is modelled for a case by 1 − *S*(*t*|*X*_*i*_) and for a control by *S*(*t*|*X*_*i*_) [13]. Akritas [16] proposed using the following model-based estimator for the conditional survival probability called the weighted Kaplan-Meier estimator and is defined by

$$ {\widehat{S}}_{\lambda_n}\left( t\Big|{X}_i\right)={\displaystyle \prod_{a\in {T}_n, a\le t}}\left\{1-\frac{{\displaystyle {\sum}_j}{K}_{\lambda_n}\left({X}_j,{X}_i\right)\boldsymbol{I}\left({Z}_j= a\right){\delta}_j}{{\displaystyle {\sum}_j}{K}_{\lambda_n}\left({X}_j,{X}_i\right)\boldsymbol{I}\left({Z}_j\ge a\right)}\right\} $$

where ${K}_{\lambda_n}\left({X}_j,{X}_i\right)$ is a kernel function that depends on a smoothing parameter *λ*_*n*_. Akritas [16] uses a 0/1 nearest neighbour kernel, ${K}_{\lambda_n}\left({X}_j,{X}_i\right)= I\left(-{\lambda}_n<{\widehat{F}}_X\left({X}_i\right)-{\widehat{F}}_X\left({X}_j\right)<{\lambda}_n\right)$ where 2*λ*_*n*_ ∈ (0, 1) represents the percentage of individuals that are included in each neighbourhood (boundaries). The resulting sensitivity and specificity are defined by

$$ \widehat{S e}\left( c, t\right) = \frac{\left(1-{\widehat{F}}_X(c)\right)-{\widehat{S}}_{\lambda_n}\left( c, t\right)}{1-{\widehat{S}}_{\lambda_n}(t)},\widehat{S p}\left( c, t\right)=1-\frac{{\widehat{S}}_{\lambda_n}\left( c, t\right)}{{\widehat{S}}_{\lambda_n}(t)} $$

where ${\widehat{S}}_{\lambda_n}(t)={\widehat{S}}_{\lambda_n}\left(-\infty, t\right)$. The above estimates of the sensitivity and specificity will produce ROC curve estimates that are invariant to monotone transformations of the marker. Both sensitivity and specificity are monotone and bounded in [0, 1]. Further, as contrast to CD1, this nonparametric method is efficient as a semi-parametric method and allows the censoring to depend on the marker space [16]. Heagerty et al. [1] used bootstrap resampling to estimate the confidence interval for this estimator. Motivated by the results gained by Akritas [16], Cai et al. [17], Hung and Chiang [2] and Hung and Chiang [18] discusses the asymptotic properties of CD2. They have established the usual $\sqrt{n}$-consistency and asymptotic normality and concluded that bootstrap resampling techniques can be used to estimate the variances. In practice, it is suggested that the value for *λ*_*n*_ is chosen to be $\mathcal{O}\left({n}^{-\frac{1}{3}}\right)$ [1]. Song and Zhou [19] extended the method to incorporate covariates other than those variables contained in the marker for constructing the ROC curves within this CD2 methodology. They have also explored their model by incorporating an ID mechanism.

#### (CD3) Kaplan-Meier like estimator of Chambless and Diao [4]

Chambless and Diao [4] highlighted the problem with the direct estimation of time-dependent sensitivity, specificity and AUC when the event status is not known at time *t* for individuals censored prior to *t*. They proposed a “Kaplan-Meier like” estimator that needs recursive computation using the riskset at each ordered event time, and mimics the Kaplan-Meier estimator. Blanche et al. [13] slightly revised the original estimation for the ease of computation. Let *t*_*k*_ be the *k* ^*th*^ observed ordered event time and *t*_*m*_ be the last observed event time before target time *t*. The sensitivity and specificity are defined by

$$ \begin{array}{l}\widehat{S e}\left( c, t\right)=\frac{{\displaystyle {\sum}_{k=1}^m} I\left({X}_{d(k)}> c\right)\left\{\widehat{S}\left({t}_{k-1}\right)-\widehat{S}\left({t}_k\right)\right\}}{1-\widehat{S}\left({t}_m\right)}\\ {}\widehat{S p}\left( c, t\right)=\frac{{\widehat{F}}_X(c)-{\displaystyle {\sum}_{k=1}^m} I\left({X}_{d(k)}\le c\right)\left\{\widehat{S}\left({t}_{k-1}\right)-\widehat{S}\left({t}_k\right)\right\}}{\widehat{S}\left({t}_m\right)}\end{array} $$

where *d*(*k*) is the index of the individual who experiences an event at time *t*_*k*_, *I*(*X*_*d*(*k*)_ > *c*) estimates *P*(*X*_*i*_ > *c*|*t*_*k* − 1_ < *T*_*i*_ ≤ *t*_*k*_) and *I*(*X*_*d*(*k*)_ ≤ *c*) estimates *P*(*X*_*i*_ ≤ *c*|*t*_*k* − 1_ < *T*_*i*_ ≤ *t*_*k*_). *Ŝ*(*t*_*k*_) is the Kaplan-Meier survival function at time *t*_*k*_ and *Ŝ*(*t*_*k* − 1_) − *Ŝ*(*t*_*k*_) estimates *P*(*t*_*k* − 1_ < *T*_*i*_ ≤ *t*_*k*_).

An advantage of this method is the sensitivity is monotone and bounded in [0, 1]. A nice property of this nonparametric estimator is that it does not involve any smoothing parameter, unlike CD2. Chambless and Diao [4] have compared CD3 with the c-statistic gained from the logistic regression model of baseline values in a simulation study and apparently it shows little bias. In order to compute variances and confidence intervals of this estimator, Chambless and Diao [4] suggested using bootstrap re-sampling.

#### (CD4) alternative estimator of Chambless and Diao [4]

CD1 estimates the conditional survival functions *S*(*t*|*X* > *c*) using the Kaplan-Meier method under the subset defined by *X* > *c*. Thus, for a large threshold value *c*, the subset for *X* > *c* may be small for estimating the conditional Kaplan-Meier estimate. However, in clinical applications, this “tail” survival function is often of interest [4]. In order to solve this problem, Chambless and Diao [4] proposed an alternative estimator, CD4, which is a model-based estimator like CD2_,_ but differs in the way of estimating the survival function. CD4 estimates the coefficients of risk factors from a Cox proportional hazards model and then these coefficients are used to estimate the survival function while CD2 uses nearest neighbour estimator of *S*(*t*|*X* > *c*). The proposed sensitivity and specificity are defined by

$$ \widehat{Se}\left( c, t\right)=\frac{E\left[\left(1- S\left( t\Big|{X}_i\right)\right) I\left({X}_i> c\right)\right]}{E\left[1- S\left( t\Big|{X}_i\right)\right]},\kern0.5em \widehat{Sp}\left( c, t\right)=\frac{E\left[ S\left( t\Big|{X}_i\right) I\left({X}_i< c\right)\right]}{E\left[ S\left( t\Big|{X}_i\right)\right]} $$

where *X* here is a score from a survival function. This estimator requires the use of a score *X* from a survival function [4] instead of the raw marker value or score from other model. So, CD4 is readily available if *X* is a score produced from a survival model but if *X* is from an external source, then we need to fit a survival model and produce the equivalent score [4]. Chambless and Diao [4] suggested estimating the conditional survival function *S*(*t*|*X*_*i*_) under a Cox model and replacing the expected values by sample means. Therefore, CD4 is immediately available at any given time. Further, CD4 also produces monotone sensitivity and specificity given the survival function holds the property that the score is produced from a survival model. Simulation study by Chambless and Diao [4] showed that CD4 is more efficient than CD3, as long as the survival model is not misspecified [20]. As with CD2, this model-based estimator also allows censoring to depend on the marker. The disadvantage of CD4 is that it is not invariant to an increasing transformation of the marker (as the score *X* from a survival function) which is a desirable property of ROC curve estimator [13] and for this reason Blanche et al. [13] choose not to compare this method to the others and the authors will not compare here too.

#### (CD5) Inverse probability of censoring weighting

CD5 was proposed by Uno et al. [21] and Hung and Chiang [18] and modifies the naïve estimator by adding weights to the observed marker values and time of disease onset in a subsample of uncensored individuals before time *t*. The weights are the probabilities of being uncensored when calculating the sensitivity:

$$ \widehat{S e}\left( c, t\right)=\frac{{\displaystyle {\sum}_{i=1}^n} I\left({X}_i> c,{Z}_i\le t\right)\left\{{\delta}_i/ n{\widehat{S}}_c\left({Z}_i\right)\right\}}{{\displaystyle {\sum}_{i=1}^n} I\left({Z}_i\le t\right)\left\{{\delta}_i/ n{\widehat{S}}_c\left({Z}_i\right)\right\}} $$

where *Ŝ*_*c*_(*Z*_*i*_) is the Kaplan-Meier estimator of the survival function of the censoring time *C*_*i*_ at the *i* ^*th*^ observed event-time *Z*_*i*_. As discussed by Blanche et al. [13], the above estimate of sensitivity is the same as in CD3 although this is not mentioned by the authors. The specificity remains the same as in the above specified naïve estimator. CD5 produces monotone sensitivity and specificity and are bounded in [0,1] [13].

#### (CD6) Conditional IPCW

CD6 is a modified version of IPCW that uses the weights that are the conditional probability of being uncensored given the marker, instead of the marginal probability of being uncensored [13]. This nonparametric estimator is robust to marker dependent censoring as previous model-based estimators CD2 and CD4. The sensitivity and specificity are estimated by

$$ \begin{array}{l}\widehat{S e}\left( c, t\right)=\frac{{\displaystyle {\sum}_{i=1}^n} I\left({X}_i> c,{Z}_i\le t\right)\left\{{\delta}_i/ n{\widehat{S}}_c\left({Z}_i\Big|{X}_i\right)\right\}}{{\displaystyle {\sum}_{i=1}^n} I\left({Z}_i\le t\right)\left\{{\delta}_i/ n{\widehat{S}}_c\left({Z}_i\Big|{X}_i\right)\right\}}\\ {}\widehat{S p}\left( c, t\right)=\frac{{\displaystyle {\sum}_{i=1}^n} I\left({X}_i\le c,{Z}_i> t\right)\left\{1/ n{\widehat{S}}_c\left( t\Big|{X}_i\right)\right\}}{{\displaystyle {\sum}_{i=1}^n} I\left({Z}_i> t\right)\left\{1/ n{\widehat{S}}_c\left( t\Big|{X}_i\right)\right\}}\end{array} $$

where *S*_*c*_(*t*|*X*_*i*_) = *P*(*C*_*i*_ > *t*|*X*_*i*_) is the censoring survival probability that may be estimated using a Cox model. However, Blanche et al. [13] suggested using the nonparametric weighted KM estimator as discussed in CD2, in order to estimate the survival function *S*_*c*_(*t*|*X*) which is also monotone and bounded in [0, 1].

#### (CD7) Weighted AUC (t)

Lambert and Chevret [5] used a similar approach to Heagerty and Zheng [12] and proposed a time-dependent weighted AUC estimator restricted to a fixed time interval (*τ*_1_, *τ*_2_) and defined as:

$$ {\widehat{AUC}}_{\omega \tau 1\tau 2}^{C, D}=\frac{1}{\widehat{S}\left(\tau 1\right)-\widehat{S}\left(\tau 2\right)}\left[{\displaystyle {\sum}_{\tau 1\le (i)\le \tau 2}{\widehat{AUC}}^{C, D}\left({t}^{(i)}\right)\left\{\widehat{S}\left({t}^{(i)}\right)-\widehat{S}\left({t}^{\left( i-1\right)}\right)\right\}}\right], $$

where *t* ^(i)^ are the ordered distinct failure times for which, if *t* ^(1)^ > *τ*_1_, it is assumed that *t* ^(0)^ = *τ*_1_, *Ŝ*(*t*), is the Kaplan-Meier estimate of the survival function and ${\widehat{AUC}}^{C, D}(t)$ is a nonparametric estimator of a C/D time-dependent AUC such as CD2or CD5 or any other estimator. The value *τ*_2_ can be allocated as the value slightly below the maximum expected follow-up time if no clinically motivated choice is specified [22]. Bootstrap resampling is used to compute the confidence intervals of CD7. Since this weighted AUC is defined under C/D, it is not directly related to concordance measures, unlike the integrated AUC that will discuss under I/D definition. However, the proposed estimator is better understood by physicians and also closer to the clinical setting since most clinical studies want to distinguish between individuals who fail and individuals who survive the disease from baseline to any particular time *t.* It is easy to implement since it can use any C/D estimators.

#### (CD8) Viallon and Latouche [20] Estimators

Viallon and Latouche [20] proposed several estimators of the time-dependent AUC relying on different estimators of the conditional absolute risk function. The conditional absolute risk function is estimated under the standard Cox proportional hazard model (VL Cox), an Aalen additive model (VL Aalen) or using the conditional Kaplan-Meier estimator (VL KM). The estimator of the time-dependent AUC is defined by

$$ AU{C}_n(t)=\frac{{\displaystyle {\sum}_{i=1}^n}\frac{i}{n}{\widehat{F}}_n\left( t;{X}_i\right)-{\left\{{\displaystyle {\sum}_{i=1}^n}{\widehat{F}}_n\left( t;{X}_i\right)\right\}}^2/2}{{\displaystyle {\sum}_{i=1}^n}{\widehat{F}}_n\left( t;{X}_i\right)\left\{1-{\displaystyle {\sum}_{i=1}^n}{\widehat{F}}_n\left( t;{X}_i\right)\right\}} $$

where *n* is the number of individuals and *X*_*k*_ denotes the *k* ^*th*^ order statistic attached to the marker *X*_1_, *X*_2_, …, *X*_*n*_. The conditional absolute risk is defined by *F*(*t*; *X* = *x*) = *P*(*T* ≤ *t*|*X* = *x*) and its estimator denoted by ${\widehat{F}}_n\left( t; X= x\right)$ is estimated as below.

*VL Cox*: Consider the Cox model [23] under the conditional hazard rate *λ*(*t*; *X* = *x*) = *λ*_0_(*t*)exp(*αx*) where *λ*_0_ denotes the baseline hazard rate, and *α* is the log hazard ratio pertaining to *X* = *x*. The conditional cumulative hazard rate of *T* = *t* given *X* is denoted by $\varLambda \left( t; X= x\right)={\displaystyle {\int}_0^t}\lambda \left( u; X= x\right) d u$. Then the estimator of the conditional absolute risk function for VL Cox is given by

$$ {\widehat{F}}_{n, Cox}\left( t; X= x\right)=1- exp\left\{-{\widehat{\varLambda}}_0(t) \exp \left(\widehat{\alpha} x\right)\right\}. $$

VL Cox is very similar to the estimator proposed by Heagerty and Zheng [12] that will be introduced in method ID1 but it does not involve the computation of the bivariate expectation [20].

*VL Aalen*: For the Aalen additive model [24], the conditional hazard rate *λ*(*t*; *X* = *x*) takes the form *β*_0_(*t*) + *β*_1_(*t*)*x*. Thus the estimator of the conditional absolute risk function for VL Aalen is given by

$$ {\widehat{F}}_{n, Aalen}\left( t; X= x\right)=1- \exp \left(-{\widehat{\beta}}_0(t)-{\widehat{\beta}}_1(t) x\right). $$

*VL KM*: A nearest-neighbour type estimator of conditional absolute risk function is used for VL KM and is defined by

$$ {\widehat{F}}_{n, KM}\left( t; X= x\right)=1-{\displaystyle \prod_{Z_{i\le t,\ {\delta}_i=1}}}\left\{\frac{K_{l_n}\left({X}_i, x\right)}{{\displaystyle {\sum}_j} I\left({Z}_j\ge {Z}_i-\right){K}_{l_n}\left({X}_j, x\right)}\right\} $$

where *l*_*n*_ is the smoothing parameter of the 0/1 symmetric nearest neighbour kernel ${K}_{l_n}$ [16].

VL estimators are straightforward to implement since they just plug-in the estimates of the conditional absolute risk function into the time-dependent AUC estimator. This plug-in nature allows their theoretical properties to follow the other established estimators of the conditional absolute risk function. Moreover, it is advisable to use CD8 compared to CD2 in the situations where the independence assumption between censoring time *C*, and the pair (*T, Z*) might be violated [20].

#### *Incident* sensitivity and *dynamic* specificity (I/D)

There are three estimation methods proposed under the I/D definition, these are discussed in ID1 – ID3 below.

Specific notation: Let *R*_*i*_(*t*) = *I*(*Z*_*i*_ ≥ *t*) denote the at-risk indicator. Let ℛ_*i*_(*t*) = (*i* : *R*_*i*_(*t*) = 1) denote the individuals that are in the riskset at time *t*, which ℛ_*t*_^1^ = (*i*; *T*_*i*_ = *t*), are individuals with the event (cases) and ℛ_*t*_^0^ = (*i*; *T*_*i*_ > *t*) are individuals without the event (controls). Let *n*_*t*_ = |ℛ_*t*_^0^| be the size of the control set at time *t* and *d*_*t*_ = |ℛ_*t*_^1^| the size of case set at time *t*. Note that the riskset at time *t* can be represented as ℛ_*t*_ = (ℛ_*t*_^1^ ∪ ℛ_*t*_^0^).

#### (ID1) Cox regression

Heagerty and Zheng [12] used the standard Cox regression model to estimate the sensitivity and specificity by the following three steps:

(i) Fit a Cox model *λ*_0_(*t*)exp(*X*_*i*_ *γ*) where $\gamma$ is the proportional hazard regression parameter. In order to relax the proportionality assumption, use a regression-smoothing method to estimate the time-varying coefficient $\widehat{\gamma}(t)$ and use it to estimate sensitivity in (ii) instead of *y*.
(ii) The sensitivity can be evaluated using $\widehat{\gamma}(t)$ from (i) as follows
  $$ \widehat{Se}\left( c, t\right)={\displaystyle {\sum}_i} I\left({X}_i> c\right){\pi}_k\left(\widehat{\gamma}(t), t\right). $$

Here *π*_*i*_(*γ*(*t*), *t*) = *R*_*i*_(*t*)exp(*X*_*i*_ *γ*(*t*))/*W*(*t*) are the weights under a proportional hazard model and *W*(*t*) = ∑_*i*_ *R*_*i*_(*t*) exp(*U*_*i*_^*T*^ *β*) with time-invariant covariates *U*_*i*_.

(iii) The specificity can be estimated empirically as follow
  $$ \widehat{Sp}\left( c, t\right)=1-{\displaystyle {\sum}_k} I\left({X}_k> c\right)\frac{{\mathrm{\mathcal{R}}}_i^0(t)}{n_t}. $$

Heagerty and Zheng [12] suggested using flexible semiparametric methods such as locally weighted maximum partial likelihood (MPL) by Cai and Sun [25] as the regression-smoothing method in (i), and simple local linear smoothing of the scaled Schoenfeld residuals [26] for reducing the bias [12].

The sensitivity is consistent for both the proportional and non-proportional hazards models whenever a consistent estimator of $\widehat{\gamma}(t)$ is used [27]. Since the specificity is an empirical distribution function calculated over the control set, it is consistent provided the control set represents an unbiased sample [12]. It is suggested that the computation of standard errors and confidence intervals is carried out using the nonparametric bootstrap based on resampling of observations (*X*_*i*_, *Z*_*i*_, *δ*_*i*_) [12].

#### (ID2) weighted mean rank

ID2 was proposed by Saha-Chaudhuri and Heagerty [28] and is based on the idea of ranking the individuals in the riskset by their respective scores. The proposed time-dependent AUC is based on local rank-based cy given time *t*, an estimator of *AUC*(*t*) is defined by

$$ A(t)=\frac{1}{n_t{d}_t}{\displaystyle \sum_{i\in {\mathrm{\mathcal{R}}}_t^1}}{\displaystyle \sum_{j={\mathrm{\mathcal{R}}}_t^0}}1\left({X}_i>{X}_j\right). $$

However, frequently, only a small number of individuals experience the event at *t*, and therefore the information on the neighbourhood is needed in order to estimate the marker concordance at *t* which is defined by

1 $$ W M R(t)=\frac{1}{\left|{\mathcal{N}}_t\left({h}_n\right)\right|}{\displaystyle \sum_{t_j\in {\mathcal{N}}_t\left({h}_n\right)}} A\left({t}_j\right) $$

where ${\mathcal{N}}_t\left({h}_n\right)=\left({t}_j:\left| t-{t}_j\right|<{h}_n\right)$ denotes a neighbourhood around *t*. This is a nearest-neighbour estimator of the AUC and can be generalized to

2 $$ \widehat{AUC}(t)={\displaystyle \sum_j}{K}_{h_n}\left( t-{t}_j\right).\  A\left({t}_j\right) $$

where ${K}_{h_n}$ is a standardized kernel function such that ${\displaystyle {\sum}_j}{K}_{h_n}\left( t-{t}_j\right)=1$. Eq. (1) is a smoothed version of Eq. (2) and it is based on local U-statistics summaries. Saha-Chaudhuri and Heagerty [28] suggested integrated mean square error (IMSE) as a potential method to select an optimal bandwidth.

Under certain conditions, Saha-Chaudhuri and Heagerty [28] showed that *WMR*(*t*) follows a normal distribution. It is suggested that this variance estimator for inference can be used in practice since it is simple and does not require resampling methods. Moreover, Saha-Chaudhuri and Heagerty [28] also provided the details of large sample properties of this estimator, and then the construction of a confidence interval for *WMR*(*t*) using the asymptotic properties is straightforward. Although it is desirable to obtain the simultaneous confidence bands for the function *WMR*(*t*), the theory may not be applicable in this case since the limiting process may not possess an independent increment structure. Instead, a simulation of a Gaussian process while keeping the estimates of ID2 fixed is needed to approximate the distribution of the Gaussian process and to estimate the quantiles. ID2 also has the advantage to be potentially robust since the relative bias remains significantly lower than for the ID1estimator.

#### (ID3) fractional polynomial

As the ID2 method is computationally intensive, especially in the selection of the bandwidth, Shen et al. [29] proposed a semi-parametric time-dependent AUC estimator which is easier and more applicable when comparing and screening a large number of candidate markers. The suggested model used fractional polynomials [30], the parameters of which are estimated by using a pseudo partial-likelihood function.

Denote *η*(.) as the link function, e.g. the logistic function. *AUC*(*t*)is modelled directly as a parametric function of time *t* using fractional polynomials of G degree:

3 $$ \eta \left( AUC(t)\right)={\displaystyle \sum_{g=0}^G}{\beta}_g{t}^{\left({p}_g\right)} $$

where for *g* = 1, …, *G*, and

$$ {t}^{\left({p}_g\right)}=\left\{\begin{array}{c}\hfill {t}^{p_g}\kern1em  if\ {p}_g\ne 0\hfill \\ {}\hfill \ln (t)\kern1.25em  if\ {p}_g=0\hfill \end{array}\right. $$

*p*_1_ ≤ … ≤ *p*_*g*_ are real-valued powers, and *β*_0_, …, *β*_*g*_ are unknown regression parameters. The choice of powers is from the set (-2, -1, -1/2, 0, ½, 1, 2) as suggested by Royston and Altman [30]. Unlike the conventional polynomial, the fractional polynomial is flexible and can mimic many function shapes in practice [30]. In order to construct the pseudo partial-likelihood, consider two types of events on each riskset *R*(*t*_*k*_) derived from the observed data which are defined by

$$ \begin{array}{l}{e}_1\left({X}_i,{X}_j,\ {Z}_i,\ {Z}_j\right)=\left\{{X}_i>{X}_j\Big|{Z}_i={t}_k,{\delta}_i=1, j\in R\left({t}_k\right)\right\}\\ {}{e}_2\left({X}_i,{X}_j,\ {Z}_i,\ {Z}_j\right)=\left\{{X}_i\le {X}_j\Big|{Z}_i={t}_k,{\delta}_i=1, j\in R\left({t}_k\right)\right\}\end{array} $$

where event *e*_1_(*X*_*i*_, *X*_*j*_, *Z*_*i*_, *Z*_*j*_) and *e*_2_(*X*_*i*_, *X*_*j*_, *Z*_*i*_, *Z*_*j*_) are respectively called a concordant and a discordant events as *e*_1_(*X*_*i*_, *X*_*j*_, *Z*_*i*_, *Z*_*j*_) occurs if individual *j* has smaller marker value than individual *i*, and *e*_2_(*X*_*i*_, *X*_*j*_, *Z*_*i*_, *Z*_*j*_) occurs if individual j has greater marker value than individual *i*, given that individual *j* has longer survival. For each event time *t*_*k*_, the counts of the two types of events are given by

$$ \begin{array}{l}{n}_1(t)={\displaystyle \sum_j} I\left\{ j:{X}_i> X\Big|{}_j{Z}_i={t}_k,{\delta}_i=1, j\in R\left({t}_k\right)\right\}\\ {}{n}_2(t)={\displaystyle {\sum}_j} I\left\{ j:{X}_i\le {X}_j\Big|{Z}_i={t}_k,{\delta}_i=1, j\in R\left({t}_k\right)\right\}.\end{array} $$

Note that at each time point *t*_*k*_, conditional on riskset *R*(*t*_*k*_), the count *n*_1_(*t*_*k*_) follows a distribution with probability equal to *AUC*(*t*_*k*_). The pseudo partial-likelihood is constructed by multiplying all probabilities of observing concordant and discordant counts over all of the risksets from the observed event times as below

$$ L\left(\beta \right)\alpha\ {\displaystyle {\prod}_{k=1}^K} A U C{\left({t}_k;\beta \right)}^{n_1\left({t}_k\right)}{\left\{1- AUC\left({t}_k;\beta \right)\right\}}^{n_2\left({t}_k\right)}. $$

Maximizing this pseudo partial-likelihood yields parameter estimates $\widehat{\beta}$. Then the time-dependent AUC estimate is obtained from Eq. (3) as a smooth function of time *t* and *β*. In practice, the integrated AUC is always of interest for the I/D definition and it can be defined by ${\displaystyle {\int}_0^{\tau}}\omega \left( t;\tau \right) A U C\left( t;\widehat{\beta}\right) d t$. When the weight function *ω*(*t*; *τ*) is invariant to time, the integrated AUC can be viewed as the global average of the AUC curve [29]. One major advantage of this estimator compared to ID2 is that the proposed method estimates the entire curve as a function of *t* and *β* while ID2 just uses a point-wise approach to estimate AUC. Further, this method is understandable and it is easier to make inference since it is a “regression-type” method, with covariates being functions of time. In estimating the integrated AUC, the ID3 method is more convenient since it uses an analytical expression while ID2 computation is more complex since the kernel-based estimation procedure has to be repeated N times, and also the selection of bandwidths has to be considered. However, Saha-Chaudhuri and Heagerty [28] decreased the computational burden by calculating the integrated AUC as an average of *AUC*(*t*) at 10 time points, which can lead to approximation errors.

#### *Incident* sensitivity and *static* specificity (I/S)

There is only one estimation method proposed under the I/S definition found from the methodological review and one extended method which are discussed below.

#### (IS1) Marginal regression modelling approach

Cai et al. [6] proposed an estimation approach using marginal regression modelling which was first proposed by Leisenring et al. [31] that accommodates censoring. Let the data for analysis be given by ((*Y*_*ik*_, ***U***_*i*_, *Z*_*i*_, *δ*_*i*_, *s*_*ik*_), *i* = 1, …, *n*; *k* = 1, …, *K*_*i*_), where ***U***_i_ denote the vector of covariates associated with *Y*_*ik*_ and let *T*_*ik*_ be the time lag between the measurement time and the event time, i.e. *T*_*ik*_ = *T*_*i*_ − *s*_*ik*_ Cai et al. [6] modelled the marginal probability associated with (*Y*_*ik*_, *T*_*ik*_, ***U***_*i*_) and the sensitivity and specificity are defined by marginal probability models,

$$ \begin{array}{l} Se\left( t,{s}_{i k},{\boldsymbol{U}}_{i,} c\right)= P\left({Y}_{i k}> c\Big|{T}_{i k}= t,{\boldsymbol{U}}_{i,}{s}_{i k}\right)={g}_D\left\{\eta {\boldsymbol{\alpha}}_0\left( t,{s}_{i k}\right)+\boldsymbol{\beta} {\boldsymbol{\hbox{'}}}_0{\boldsymbol{U}}_i+{h}_0(c)\right\}\\ {} Sp\left({t}^{*},{s}_{i k},{\boldsymbol{U}}_{i,} c\right)= P\left({Y}_{i k}\le c\Big|{T}_{i k}>{t}^{*},{\boldsymbol{U}}_{i,}{s}_{i k}\right)=1-{g}_{\overline{D}}\left\{\xi {\boldsymbol{\alpha}}_0\left({s}_{i k}\right)+\boldsymbol{b}{\boldsymbol{\hbox{'}}}_0{\boldsymbol{U}}_i+{c}_0(c)\right\}\end{array} $$

where *g*_*D*_ and ${g}_{\overline{D}}$ are specified inverse link functions, *h*_0_ and *c*_0_ are baseline functions of the threshold *c* that are completely unspecified. These nonparametric baseline functions of *c* represent the shape and location of the sensitivity and specificity functions while the parameters ***β***_0_ and ***b***_0_ quantify the covariate effects on them and *η* ***α***_0_ and $\xi$ ***α***_0_ are the time effects. The dependence on time for sensitivity is through the parametric functions *η* ***α***_0_(*t*, *s*) = ***α*** *'*_0_ ***η***(*t*, *s*) and *ξ* ***α***_0_(*s*_*ik*_) = ***α*** *'*_0_ *ξ*(*s*) where ***η*** and *ξ* are vectors of polynomial or spline basis functions.

Let **Ψ**_0_ = (**Η**_0_(.) = [*h*_0_(.), *c*_0_(.)] *'*, ***θ***_0_ = [***α*** *'*_0_, ***β*** *'*_0_, ***α*** *'*_0_, ***b*** *'*_0_]) denote all unknown parameters. Cai et al. [6] considered the marginal binomial likelihood function based on the binary variable *I*(*Y*_*ik*_ ≥ *c*) and it is defined by

$$ {\displaystyle {\prod}_{i=1}^n}{\displaystyle {\prod}_{k=1}^K}{\left\{{p}_{i k}\left( y;\boldsymbol{\Psi} \right)\right\}}^{I\left({Y}_{i k}\ge c\right)}{\left\{1-{p}_{i k}\left( y;\boldsymbol{\Psi} \right)\right\}}^{I\left({Y}_{i k}< c\right)} $$

and the corresponding score equation is solved to estimate the nonparametric baseline functions, **Η**_0_(*c*). Further, ***θ***_0_ is estimated by solving the integration of the corresponding score equation. Cai et al. [6] also proposed an approach that ignores censored observations.

Simulation studies [6] showed that the above method provides reasonably unbiased estimates of model parameters of sensitivity and specificity. The approach which includes the censored observations is always more precise than the one that excludes the censored observations.

#### (IS2) extended Cox regression

The main difference between I/D and I/S definitions is related to the controls. The controls in I/D are changing based on the target time whereas in I/S, controls are static survivors beyond a fixed time. This difference has motivated us to extend the Cox Regression method (ID1) to incorporate a longitudinally repeated marker using the I/S definition. A marker value at a particular visit time *s* is considered. Thus, we have changed the definition of the riskset as those individuals beyond target time by including those beyond a fixed follow-up. However, as I/S is not based on classification of the riskset at time *t* like I/D, this extended method cannot be said as a natural companion to hazard models. We have also extended the current software of ID1 (see Section for **Software** below) by redefining the riskset according to the I/S definition. The extended software can also be used with the baseline value of the marker.

### Additional methods for longitudinal outcomes

Three estimation methods have been proposed for a longitudinal marker in addition to those described above under I/S definition, although some do not incorporate censoring. These estimation methods are discussed below. An extension of the C/D definition for a longitudinally repeated marker is suggested as a fourth method.

Specific notation: Let $n={n}_D + {n}_{\overline{D}}$ denote the total number of individuals which is the summation of the where *n*_*D*_ is the total number of cases and ${n}_{\overline{D}}$ is the total number of controls. Let ***U***_*ik*_^*T*^ = *vec*(*T*_*i*_, *s*_*ik*_) = ***U***_*D*_ denote the vector of covariates associated with *U*_*ik*_. The total number of longitudinally repeated marker values for cases is ${N}_D={\displaystyle {\sum}_i^{n_D}{K}_i}$. The time prior to an event is defined as the time lag between the measurement time and the event time: *T*_*ik*_ = *T*_*i*_ − *s*_*ik*_ as above. Similarly for controls, let *Y*_*jl*_ be the biomarker value obtained from individual *j* at the *l* ^*th*^ visit time *s*_*jl*_ with $j={n}_D+1, \dots,\ {n}_D+{n}_{\overline{D}}$ and *l* = 1, …, *L*_*j*_. Let ${\boldsymbol{U}}_{jl}^T= v e c\left({s}_{jl}\right)={\boldsymbol{U}}_{\overline{D}}$ denote the vector of covariates associated with *Y*_*jl*_. The total number of longitudinally repeated marker values for controls is ${N}_{\overline{D}}={\displaystyle {\sum}_j^{n_{\overline{D}}}}{L}_j$. Thus, the total number longitudinally repeated marker values in study is $N={N}_D+{N}_{\overline{D}}$.

#### (AD1) Linear mixed-effect regression model

Etzioni et al. [32] proposed the use of a linear random-effect regression model of serial marker measurements as a function of time prior to event, which was originally proposed by Tosteson and Begg [33] by using ordinal regression models in order to estimate the time-dependent ROC curve statistics. This approach involves modelling the marker values and uses the model parameter estimates to induce an ROC curve at a particular time. The ROC is defined by

4 $$ ROC\left( t, p\right)={S}_D\left[{a}_0(t)+{a}_1(t){S}_{\overline{D}}^{-1}(p)\right] $$

where *t* is the time prior to event, *p* is the false positive rate, *Sp* is one minus the cumulative distribution function for cases and ${s}_{\overline{D}}$is one minus the cumulative distribution function for controls. Suppose cases and controls are from the same location-scale family *S*,*μ*_*D*_ and *S*_*D*_ are the mean and standard deviation of *Y*_*ik*_ *,* and ${\mu}_{\overline{D}}$ and ${s}_{\overline{D}}$ are the mean and standard deviation of *Y*_*jl*_. Then α_0_(*t*) and α_1_(*t*) are defined by

$$ \begin{array}{l}{a}_0(t)=\frac{\mu_{\overline{D}}-{\mu}_D}{s_D}\\ {}{a}_1(t)=\frac{s_{\overline{D}}}{s_D}.\end{array} $$

To estimate α_0_(*t*) and α_1_(*t*), Zheng and Heagerty [9] fitted the following linear mixed effect models for cases and controls:

5 $$ Case:\ {Y}_{ik}={b}_{0 i}+{b}_{1 i}{s}_{ik}+{\beta}_0+{\beta}_1{s}_{ik}+{\beta}_2{T}_{ik}+{\beta}_3{s}_{ik}{T}_{ik}+{\varepsilon}_{ik} $$

6 $$ Control:\ {Y}_{jl}={b}_{0 j}+{b}_{1 j}{s}_{jl}+{\beta}_0+{\beta}_1{s}_{jl}+{\varepsilon}_{jl} $$

Where *ε*_*ik*_ ~ *N*(0, *σ*_*D*_^2^) and (*β*_0_, *β*_1_, *β*_2_, *β*_3_) ~ *N*[(*β*_0_^*D*^, *β*_1_^*D*^, *β*_2_^*D*^, *β*_3_^*D*^), ***V*** ^*D*^] for cases and ${\varepsilon}_{jl} \sim N\left(0,{\sigma}_{\overline{D}}^2\right)$ and $\left({\beta}_0,{\beta}_1\right) \sim N\left[\left({\beta}_0^{\overline{D}},{\beta}_1^{\overline{D}}\right),{V}^{\overline{D}}\right]$ for controls. ***V*** ^*D*^. and ${\boldsymbol{V}}^{\overline{D}}$ are variance-covariance matrices for cases and controls respectively. Of note, only Eq. (5) includes the time prior to event (*T*_*ik*_) but not Eq. (6) since controls are those individuals who do not experience the event. Parameter estimates from Eqs. (5) and (6) are used to induce the ROC estimates in Eq. (4) using estimated α_0_(*t*) and α_1_(*t*). For a given *s* and *t*, *μ*_*D*_,${\mu}_{\overline{D}}$, *s*_*D*_ and ${s}_{\overline{D}}$ are estimated by

$$ {\widehat{\mu}}_D={\boldsymbol{U}}_D{\boldsymbol{\beta}}^D,{\widehat{\mu}}_{\overline{D}}={\boldsymbol{U}}_{\overline{D}}{\boldsymbol{\beta}}^{\overline{D}},{\widehat{s}}_D=\sqrt{\sigma_D^2+{\boldsymbol{U}}_D{\boldsymbol{V}}^D{\boldsymbol{U}}_D^T}\;\mathrm{and}\;{\widehat{s}}_{\overline{D}}=\sqrt{\sigma_D^2+{\boldsymbol{U}}_{\overline{D}}{\boldsymbol{V}}^{\overline{D}}{\boldsymbol{U}}_{\overline{D}}^T} $$

where $$ {\boldsymbol{U}}_{\boldsymbol{D}}=\left[\begin{array}{ccc}\hfill 1\hfill & \hfill \boldsymbol{s}\hfill & \hfill \begin{array}{cc}\hfill \boldsymbol{t}\hfill & \hfill \boldsymbol{s}\boldsymbol{t}\hfill \end{array}\hfill \end{array}\right],{\boldsymbol{\beta}}^{\boldsymbol{D}}={\left[\begin{array}{ccc}\hfill {\widehat{\boldsymbol{\beta}}}_0\hfill & \hfill {\widehat{\boldsymbol{\beta}}}_1\hfill & \hfill \begin{array}{cc}\hfill {\widehat{\boldsymbol{\beta}}}_2\hfill & \hfill {\widehat{\boldsymbol{\beta}}}_3\hfill \end{array}\hfill \end{array}\right]}^{\boldsymbol{T}},{\boldsymbol{U}}_{\overline{\boldsymbol{D}}} = \left[\begin{array}{cc}\hfill 1\hfill & \hfill \boldsymbol{s}\hfill \end{array}\right] $$ and $$ {\boldsymbol{\beta}}^{\overline{\boldsymbol{D}}}={\left[\begin{array}{cc}\hfill {\widehat{\boldsymbol{\beta}}}_0\hfill & \hfill {\widehat{\boldsymbol{\beta}}}_1\hfill \end{array}\right]}^{\boldsymbol{T}}. $$

#### (AD2) Model of ROC as a function of time prior to disease

Pepe [34] proposed the use of a regression model for the ROC curve itself, and similarly Etzioni et al. [32] proposed using a ROC model directly as a function of time prior to event. The model is defined by

$$ ROC\left( t, p\right)=\Phi \left[{\gamma}_0+{\gamma}_1{\Phi}^{-1}(p)+\alpha t\right] $$

where *p* is the false positive rate, Φ is one minus the normal cumulative distribution function. At each time *t*, it is assumed that the ROC is of the binormal form as in Eq. (4) and the ROC curves at different *t* are related through a linear effect on the intercept. In terms of (4), *a*_0_(*t*) = *γ*_0_ + *αt* and *a*_1_(*t*) = *γ*_1_. The parameters *γ*_0_,*γ*_1_ and *α* can be estimated by the following steps

(i) Construct a dataset of {(*Y*_*ik*_, *Y*_*jl*_), *D* = *I*(*Y*_*ik*_ ≥ *Y*_*jl*_)}
(ii) Calculate the quantile *p* in the control population (control observations in each pair as defined in step 1 above). It can be estimated by the empirical cumulative distribution function in the control sample.
(iii) The indicator *I*(.) in step 1 is estimated conditional on *p* in step 2. Thus, the *ROC*(*p*) is estimated by fitting a generalized linear model to data *I*(.), where the family is binomial, the link is probit and the covariates are Φ^-1^(*p*) and *T*_*ik*_.

There are a few advantages of this method compared to the first method in which the range of setting of this method is much broader [34]; the range of models that allowed for the ROC curve is broader; the model can include the interactions between *p* and U; the distributions of the test result in cases and controls do not need to be derived from the same family. Indeed, no assumptions are made regarding the distribution of marker for controls but only on the relationship between cases and controls which made through the ROC curve model.

#### (AD3) Semi-parametric regression quantile estimation

Zheng and Heagerty [9] proposed a semi-parametric regression quantile approach which is an extension to the parametric approach of Heagerty and Pepe [35] to construct time-dependent ROC curves. The definition of the ROC curve at time *t* has the same form as Eq. (4) but since in [9], the positive test is defined as a marker value less than *c*, thus true positive is defined in terms of the cumulative distribution function instead of the survival function. The ROC curve at time *t* is estimated by the conditional empirical quantile function of *Y*_*ik*_|*U*_*ik*_, as from a location-scale family and defined as follow:

$$ ROC\left( t, p\right)= F\left[{a}_0(t)+{a}_1(t){G}^{-1}(p)\right] $$

where *F* and *G* are the baseline distribution functions of case and control models as follow

$$ \begin{array}{l} Case:\ {Y}_{ik}={\mu}_D\left({\boldsymbol{U}}_{ik}\right)+{\sigma}_D\left({\boldsymbol{U}}_{ik}\right){\epsilon}_D\left({\boldsymbol{U}}_{ik}\right)\\ {} Control:\ {Y}_{jl}={\mu}_{\overline{D}}\left({\boldsymbol{U}}_{jl}\right)+{\sigma}_{\overline{D}}\left({\boldsymbol{U}}_{jl}\right){\epsilon}_{\overline{D}}\left({\boldsymbol{U}}_{jl}\right)\end{array} $$

where *μ*_*D*_ *σ*_*D*_, ${\mu}_{\overline{D}}$ and ${\sigma}_{\overline{D}}$ are the location and scale functions. Instead of using a quasi-likelihood method to estimate *μ*_*D*_ *,σ*_*D*_, ${\mu}_{\overline{D}}$ and ${\sigma}_{\overline{D}}$ [35], Zheng and Heagerty [9] used regression splines. In order to estimate the conditional baseline distribution function *F* and *G*, Zheng and Heagerty [9] proposed using an empirical distribution function of the standardized residuals if the baseline functions are independent of covariates, and to consider the symmetrized nearest neighbour (SNN) estimator [36] if the baseline functions are smooth functions of covariates. Thus, this semi-parametric estimation method gives greater flexibility than the parametric method [32] by allowing separate model choices for each of the key distributional aspects.

#### (AD4) Cumulative/Dynamic definition extending for a longitudinal marker

Zheng and Heagerty [14] proposed a generalization of CD1 by Heagerty et al. [1] for longitudinal marker measurements. The key idea was the same as for the IS2 method in which the most recent marker is used to discriminate between cases prior to time *t* from controls after time *t*. Contrasted with CD1, it is no longer just the baseline marker or prognostic information that will be used but the updated information. The proposed sensitivity and specificity take the same form of CD1. In order to estimate the distribution function ${\widehat{F}}_Y(c)$(see CD1), Zheng and Heagerty [14] used the semi-parametric regression quantile method for longitudinal data [35]. For the bivariate survival function, *S*(*c*, *t*), and the marginal survival function, *S*(*t*), Zheng and Heagerty [14] used a partly conditional hazard model as proposed by Zheng and Heagerty [37].

Motivated by the above methodology, we have extended CD2 to incorporate the most recent marker value instead of baseline marker value. CD2 is chosen rather than CD1 because CD1 produces non-monotone sensitivity or specificity. The sensitivity and specificity are defined the same as CD2. This extended CD2 (denoted as ECD2) is assumed to have all the advantages of CD2 with an extra advantage of using the most recent marker value which is more reliable in depicting current status of an individual.

### Software

The current software for computing the time-dependent ROC curves are available as R packages. These are briefly described below.

#### survivalROC

The survivalROC [38] package estimates CD1and CD2. The R documentation includes worked examples using the built-in dataset called *mayo* (Primary Biliary Cirrhosis (PBC) dataset from Mayo Clinic). The estimators can be chosen by the type of method “KM” or “NNE” in the function syntax.

#### survAUC

The package [39] provides a variety of functions to estimate time-dependent true/false positive rates and AUC for censored data. The AUC.cd can be used to calculate CD4 and it is restricted to Cox regression. The estimates obtained from this function are valid as long as the Cox model is specified correctly. The values returned by this function are AUC, integrated AUC and times at which the AUC are evaluated.

#### timeROC

The package [40] provides the functions to compute confidence intervals of AUC and tests for comparing AUC of two markers measured on the same individuals. Both CD5 and CD6 estimators can be computed by this package. It is also capable of allowing for competing risks event times.

#### survival, timereg and prodlim

The Basehaz function in the “survival” package [41] in R is used to obtain the VL Cox estimates which uses the baseline hazard under a Cox model. The Aalen function in the “timereg” package [42] can be used to estimate the conditional absolute risk under VL Aalen; it returns estimated coefficients *β*_0_ and *β*_1_. The VL KM estimator can be computed using the “prodlim” package [43]. For the selection of the smoothing parameter *l*_*n*_, a direct plug-in method can be used by setting *l*_*n*_ to 0.25 *n* ^− 1/5^.

#### risksetROC

This risksetROC package [44] estimates the time-dependent ROC curves under I/D definition and produces accuracy measures for censored data under proportional or non-proportional hazard assumption of ID1 estimator.

## Results

### Examples of applications

Among the three definitions for sensitivity and specificity, C/D has been the most commonly applied in clinical papers (69/308, 22%). The I/D definitions have been applied in 14 papers (4.6%) while none was found for the I/S definitions. The detail on the review strategy is presented as a CONSORT diagram (Additional file 1: Fig. S1A) with a brief description of the process and the discussion about the remaining papers. Since the publication by Heagerty and Zheng [12] who introduced the three definitions, the number of clinical papers that used an I/D methodology has been increased (Additional file 1: Fig. S1B). Lung, breast and liver cancer are the most common areas for the application of C/D and I/D (Additional file 1: Fig. S1C). Some of the applications of C/D and I/D from cancer are described below.

Lu et al. [45] aimed to determine a robust prognostic marker for tumour recurrence as 30% of Stage I non-small cell lung cancer (NSCLC) patients will experience the tumour recurrence after therapy. They used time-dependent ROC curve analysis to assess the predictive ability of gene expression signatures. The recurrence-related genes were identified by performing a Cox proportional hazards analysis. A 51-gene expression signature was validated as highly predictive for recurrence in Stage I NSCLC with AUC values greater than 0.85 from baseline up to 100 months of follow-up. The highest AUC values have been seen after 60 months to 100 months of follow-up with *AUC*(*t*) = 0.90, implying the 51-gene expression signature is a better marker in discriminating between Stage 1 NSCLC patients who will experience tumour recurrence up to 60 months and patients who will not experience tumour recurrence beyond 60 months of follow-up. Lu et al. [45] concluded that this gene expression signature has important prognostic and therapeutic implications for the future management of these patients.

Tse et al. [46] has developed a prognostic risk prediction model for silicosis among workers exposed to silica in China using a Cox regression analysis to screen the potential predictors. The score from this model was then developed as a unique score system which includes 6 covariates: age at entry, mean concentration of respirable silica, net years of dust explore, smoking illiteracy and number of jobs. This score system was regarded as accurate in discriminating the workers with silicosis and healthy workers up to 600 months of follow-up since the AUC values are more than 0.80. These AUC values seems to decrease from baseline *AUC*(*t* = 0) = 0.96 to the end of follow-up *AUC*(*t* = 600) = 0.83 which indicates the discrimination potential of baseline score is diminished across study follow-up. This study provides scientific guidance to the clinicians to identify high-risk workers.

Yue et al. [47], [48] have used pre-treatment 18 F-FDG-PET/CT imaging and combinatorial biomarkers respectively to stratify the risk of TNBC (Triple-negative breast cancer) patients. TNBC is considered as a high risk disease and normally associated with poor survival. A stratification of prognosis of this disease can help in identifying the patients with good prognosis for less aggressive therapy. The event-time outcome of the studies was defined as the time to recurrence from TNBC disease. Time-dependent ROC curve was used to assess the prognostic value of the biomarkers, EFGR and CK5/6 at different cut-off points and the optimal cut-off was obtained based on the AUC values. The cut-off values were estimated by maximizing both sensitivity and specificity of the event-time outcome. The optimal values of 15% with AUC = 0.675 and 50% with AUC = 0.611 for EFGR and CK5/6 were found respectively. AUC values obtained were used as a basis of a decision rule. By using the optimal cut-off value, the patients were stratified into two different risk level groups which helps in selecting the appropriate treatment strategies for patients.

Desmedt et al. [49] have studied the performance of the gene expression index (GGI) in predicting relapses in postmenopausal women who were treated with tamoxifen (T) or letrozole (L) within the BIG 1-98 trial. The predictive ability of GGI was estimated using time-dependent AUC and has been plotted as a function of time to characterize temporal changes in accuracy of the GGI marker. They have calculated *AUC*(*t* = 24) = 0.73 which implies that 73% of the patients who relapse at 24^th^ month have greater GGI score than patients who relapse after 24^th^ month. Further, *AUC* at = 27) was found to be the highest which indicates that the maximal discrimination occurs near the median follow-up time.

George et al. (2012) aimed to determine the predictive ability of lesions texture along with traditional features in order to detect the early tumour response. Texture features are important in detecting the progression of tumour among cancer patients, e.g. s (18)F-fluorodeoxyglucose (FDG) followed with positron emission tomography (PET) estimates. The event-time outcome was defined as the time to tumour progression, which is the distance between subspaces from baseline scan and follow-up scan. Time-dependent ROC curve is used to obtain the predictive ability of the weighted subspace-subspace distance from the baseline and the follow-up scan as a marker for predicting early tumour response. In a study of 15 patients who had metastatic colorectal cancer, the follow-up scan was taken at the first week after the first dose of the treatment. As a result, a concordance summary of 0.68 is found from the predictive model using weighted subspace-subspace distance metrics. This result helps as an added value in using textural information for therapy response evaluation.

### Illustrative application

We have used sequential data from a randomized placebo-controlled trial of the drug D-penicillamine (DPCA) for the treatment of primary biliary cirrhosis (PBC) conducted at the Mayo Clinic between 1974 and 1984 [50] in order to illustrate the performance of the current methods in estimating the time-dependent ROC curves. The event-time outcome of this study is the time to death due to PBC liver disease. The original clinical protocol for the study specified visits at 6 months, 1 year, and annually thereafter. We use a model score estimated from the Cox model containing five covariates: log(bilirubin), albumin, log(prothrombin time), edema and age as the marker [12].

Table 2 shows the estimated AUC from several methods at Year 1, Year 5 and Year 10 based on the baseline value of the marker or the most recent value. All methods show a decreasing of AUCs as the prediction time is further from marker measurement time. This evidenced the hypothesis we had earlier that the discriminative power of the marker becomes weaker with increasing prediction time. The methods involving longitudinal marker measurements assume that the marker value which is closest to the prediction time is better in discriminating between the cases and controls. ECD2 (discussed in AD4) used the last value prior to each prediction time produces higher values of AUC than CD2 with baseline marker measurement. This is also true for IS2 which uses the last marker prior to each prediction time and has higher AUC values than the IS2 which uses baseline marker measurement. The methods involving a longitudinal marker are usually interpreted with respect to the time lag between the last visit time and the prediction time since each individual may have a different set of visit times. Thus, the AUC values are produced in a matrix when a longitudinal marker is referred and uses the last value prior to each prediction time in the estimation. As the time lag gets longer, the AUC decreases due to the same reason with the baseline value of a marker. The R software described previously was used to estimate these models.

**Table 2:** Estimated time-dependent AUC for Year 1, Year 5 and Year 10

Definitions	Marker time	Method	AUC (SD)		
			Year 1	Year 5	Year 10
C/D	*t* = 0	Naïve	0.846 (0.023)	0.885 (0.022)	0.883 (0.030)
		CD1	0.922 (0.041)	0.921 (0.021)	0.878 (0.027)
		CD2	0.895 (0.056)	0.897 (0.024)	0.869 (0.028)
		CD3	0.922 (0.042)	0.917 (0.020)	0.898 (0.031)
		CD5	0.922 (0.042)	0.915 (0.021)	0.866 (0.028)
		CD6	0.922 (0.038)	0.915 (0.020)	0.870 (0.030)
C/D	Last value prior to:	ECD2			
	Year 1		0.926 (0.039)	0.918 (0.019)	0.871 (0.027)
	Year 5		-	0.911 (0.019)	0.910 (0.021)
	Year 10		-	-	0.899 (0.022)
I/D	*t* = 0	ID1	0.845 (0.010)	0.791 (0.028)	0.692 (0.024)
		ID3	0.893 (0.048)	0.757 (0.041)	0.716 (0.143)
I/S	*t* = 0	IS2	0.939 (0.025)	0.836 (0.028)	0.698 (0.034)
I/S	Last value prior to:	IS2			
	Year 1		0.968 (0.003)	0.872 (0.024)	0.698 (0.043)
	Year 5		-	0.957 (0.003)	0.698 (0.031)
	Year 10		-	-	0.768 (0.038)

The AD1 method used all available longitudinal marker values for prediction of time-dependent ROC curves. We fit the following models for case and controls:

$$ \begin{array}{l} Case:\ {Y}_{ik}={b}_{0 i}+{b}_{1 i} V{T}_{ik}+{\beta}_0+{\beta}_1 V{T}_{ik}+{\beta}_2 TB{E}_{ik}+{\beta}_3 V{T}_{ik} TB{E}_{ik}+{\varepsilon}_{ik},\\ {} Control:\ {Y}_{jl}={b}_{0 j}+{b}_{1 j} V{T}_{jl}+{\beta}_0+{\beta}_1 V{T}_{jl}+{\varepsilon}_{jl},\end{array} $$

where VT and TBE are longitudinal visit time and time before event respectively. The parameter estimates from the two above models are given in Table 3 below. Say we want to estimate the time-dependent ROC curve at five years prior to death i.e. *t* = 5 for the marker measured at visit time equal to ten years (*i. e. s* = 10), the means and standard deviations for cases and controls are estimated by ${\widehat{\mu}}_D={\boldsymbol{U}}_D\ {\boldsymbol{\beta}}^D=0.373$, ${\widehat{\mu}}_{\overline{D}}={\boldsymbol{U}}_{\overline{D}}\ {\boldsymbol{\beta}}^{\overline{D}}=0.492$, ${\widehat{s}}_D=\sqrt{\sigma_D^2+{\boldsymbol{U}}_D\ {\boldsymbol{V}}^D\ {\boldsymbol{U}}_D^T}=1.207$, where $$ {\boldsymbol{V}}^{\overline{D}}=\left[\begin{array}{cc}\hfill {(0.593)}^2\hfill & \hfill -7.730\times {10}^{-5}\hfill \\ {}\hfill -7.730\times {10}^{-5}\hfill & \hfill {\left(3.448\times {10}^{-4}\right)}^2\hfill \end{array}\right] $$and ${\hat{s}}_{\overline{D}}=\sqrt{\sigma_{\overline{D}}^2+{\boldsymbol{U}}_{\overline{D}}\ {\boldsymbol{V}}^{\overline{D}}\ {\boldsymbol{U}}_{\overline{D}}^T}=1.217$where $$ {\boldsymbol{V}}^{\overline{D}}=\left[\begin{array}{cc}\hfill {(0.550)}^2\hfill & \hfill 3.004\times {10}^{-5}\hfill \\ {}\hfill 3.004\times {10}^{-5}\hfill & \hfill {\left(2.615\times {10}^{-4}\right)}^2\hfill \end{array}\right] $$.

**Table 3:** Parameter estimates for linear mixed effect model

		Case	Control
Fixed Effect	${\widehat{\beta}}_0(SE)$	1.139(8.865 × 10^-2^)	-0.569 (0.043)
	${\widehat{\beta}}_1(SE)$	-4.813 × 10^-4^(4.419 × 10^-5^)	2.906 × 10^-4^ (2.502 × 10^-5^)
	${\widehat{\beta}}_2(SE)$	2.283 × 10^-4^(5.696 × 10^-5^)	
	${\widehat{\beta}}_3(SE)$	-1.083 × 10^-7^(1.605 × 10^-8^)	
Random Effect	${\widehat{\sigma}}_{int}$	0.593	0.550
	${\widehat{\sigma}}_{VT}$	3.448 × 10^-4^	2.615 × 10^-4^
	${\widehat{\rho}}_{int, VT}$	-0.378	0.209
	${\widehat{\sigma}}_{Res}$	0.293	0.220

**Fig. 2:**
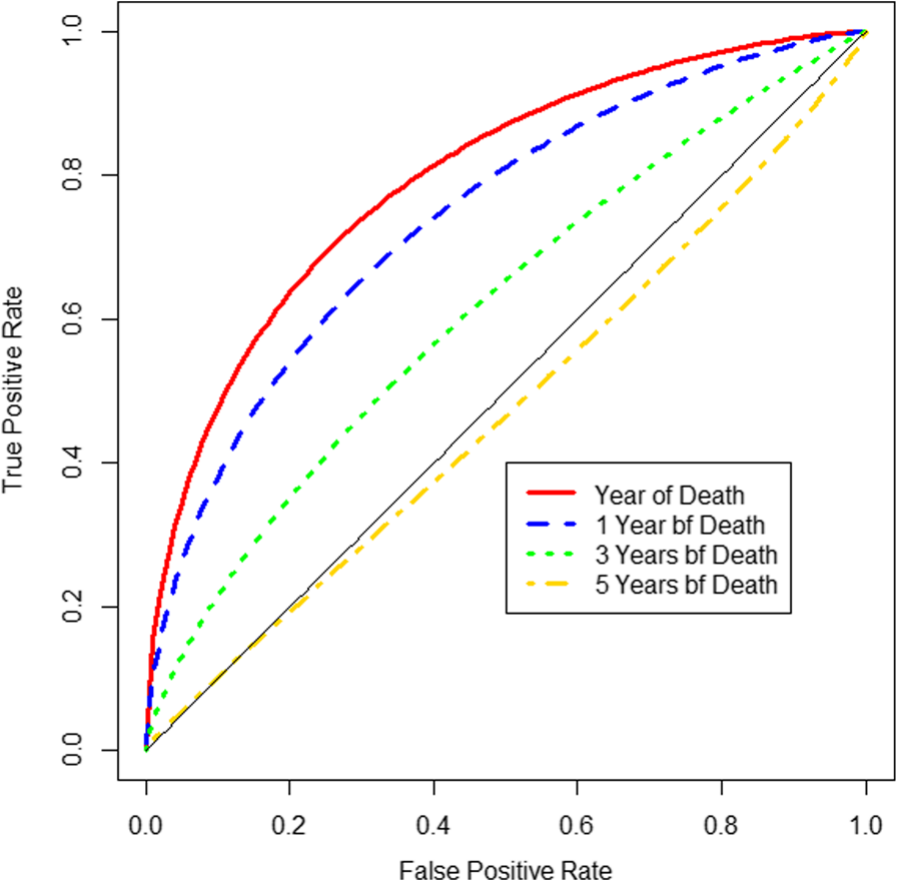
Time-dependent ROC curves for 0, 1, 3, 5 years prior to death for the marker measured at visit time at ten years

The corresponding ROC curves are shown in Fig. 2 for 0, 1, 3 and 5 years prior to death at visit time at 10 years (year 0 implies that the death is occurred at 10 years since enrolment to the study). Figure 2 clearly shows that the discrimination is better when the marker is measured at times closer to death. The estimated AUC value for five years before death is about 0.5, hence it can be concluded that the marker is useless to be used for discrimination between cases and controls at five years before death.

## Discussion and conclusions

Although C/D is the most commonly being applied, if a researcher has a specific time point of interest in order to distinguish between individuals with an event and individuals without event at that time point, I/D or I/S is more appropriate. Since I/S requires a fixed follow-up to observe the clinical outcome of interest, it can be applied in long follow-up studies with longitudinally measured markers. C/D and I/D are usually used for a single biomarker value while I/S can include a longitudinal biomarker. As the disease status of an individual may change during follow-up, the biomarker values may also change, and hence, the most recent marker value may be best related to the current disease status of an individual. Thus, usage of the most recent marker value prior to a target prediction time *t* is acceptable as we discussed using the extended methods.

The optimal cut-off is determined by choosing the highest AUC value in which describes the marker has the largest separation between cases and controls. In general, the cut-off (also called as threshold) is chosen based on the availability of the healthcare resources and the level of disease severity.

None of the methods discussed earlier used a complete history of longitudinal marker conditional on an event-time. The approach of considering a more complete record of each individual when estimating the ROC summaries over time can be more appropriate. A joint modelling framework in an attempt to estimate the time-dependent ROC curve is recommended since it considers the association between longitudinal marker and the corresponding event-time processes. Further, it is also suggested to assume the event times to be parametrically distributed which is then be easier to estimate the survival function if a researcher is attempting to extend for measurement error.

## Additional file

Additional file 1: S1, Review strategy and additional results, the detail of the comprehensive review strategy with description of the process, additional results and references from the review. (DOCX 1615 kb)

## Abbreviations

18 F-FDG-PET/CT: Fluorine-18-fluorodeoxyglucose positron emission tomography/computed tomography
AIDS: Acquired immune deficiency syndrome
AUC: Area under ROC curve
C/D: Cumulative/Dynamic
CD4: T–lymphocyte cell bearing CD4 receptor
DPCA: Drug D-penicillamine
FDG: F-fluorodeoxyglucose
GGI: Gene expression index
HIV: Human immunodeficiency virus
I/D: Incident/Dynamic
I/S: Incident/Static
NSCLC: Non-Small Cell Lung cancer
OCT: Optical coherence tomography
PBC: Primary biliary cirrhosis
PET: Positron emission tomography
RNFL: Retinal nerve fiber layer
ROC: Receiver operating characteristic
TNBC: Triple-negative breast cancer
