# Supplementary material for: Time-dependent ROC curve analysis in medical research: current methods and applications
Source: BMC Med Res Methodol. 2017 Apr 7;17:53. doi: 10.1186/s12874-017-0332-6 (PMC5384160; doi:10.1186/s12874-017-0332-6)
Supplement: Additional file 1: — S1, Review strategy and additional results, the detail of the comprehensive review strategy with description of the process, additional results and references from the review. (DOCX 1615 kb) [file 12874_2017_332_MOESM1_ESM.docx]

Additional file 1 for “Time-dependent ROC curve analysis in medical research: current methods and applications”

**Adina Najwa Kamarudin ^*^, Trevor Cox, Ruwanthi Kolamunnage-Dona**

# Review strategy and additional results

We have used combinations of keywords including ROC, AUC, time-dependent, time-specific and accuracy in order to find the relevant papers for our review. The number of papers we had in each search from MEDLINE (Ovid) is reported in Table A below. Some of the papers found were duplicating but Table A only presents the results after removed the duplicates.

**Table S1A Results from MEDLINE (Ovid)**

| Search | Keyword | Limitation | Results |
| --- | --- | --- | --- |
| 1 | ROC and Time-dependent | Abstract | 108 |
| 2 | ROC and Time-dependent | Title | 0 (duplicates) |
| 3 | ROC and Time-specific | Abstract | 2 |
| 4 | AUC and Time-dependent | Abstract | 208 |
| 5 | AUC and Time-dependent | Title | 2 |
| 6 | Accuracy and Time-dependent | Title | 4 |
| TOTAL | | | 324 |

We reviewed title or abstract of each paper to decide whether it is related to methodology of time-dependent ROC curve or only clinical applications. We have also used Scopus search with the same keywords and found extra ten papers which resulted in a total of 334 papers. The details of the review process are described in Figure A below.

Included (n=16)

Total

**Identification**

**Screening**

**Eligibility**

**Include**

Papers identified through searching from MEDLINE (Ovid) and Scopus (n=324+8=332)

Papers identified through searching from the internet (n=5) and list of reference (n=6)

Included in methodological review (n=24)

After screening based on title and abstract

Applications (n=308)

Reasons:

1. Time-dependent ROC; C/D only (n=69) [16-84], I/D only (n=14)[85-98], both C/D and I/D (n=2) [14, 15, 99, 100], Others (n=2) [101, 102], Unclear (n=5) [103-107]
2. Standard ROC (n=216) [108-323]

Included (n=35)

After read the full-text for eligibility

Included (n=16)

Within the scope of review

Excluded (n=19)

Reasons:

1. Competing risks (n=4)
2. Reviews (n=3)
3. Bayesian (n=2)
4. Sample size determination (n=1)
5. Missing biomarker (n=1)
6. Discriminant index (n=1)
7. Diverse censoring (n=1)
8. Censored predictor (n=1)
9. Microarray data (n=1)
10. Others (n=4)

Cumulative/Dynamic (n=10) [2, 4-12]

Incident/Dynamic (n=3) [1-3]

Incident/Static or Others

(n=3) [13-15]

**Extraction**

**Figure S1A CONSORT diagram**

Among the total of 332 papers, only 24 papers included in methodological review and the remaining 308 papers were the clinical application papers which used standard or time-dependent ROC curves analyses. In order to ensure the relevance of these 24 methodological papers, the full-text of each paper was assessed. We also searched the internet by using the same keywords. Five further papers were found through internet search and four papers were found after checking the list of references in each paper. This resulted in 35 papers eligible for the methodological review. If there were any ambiguities or confusion as to the extracted data, the second and third authors were consulted. They were 19 papers were excluded then because beyond the scope of this review. Out of the final 16 papers, 10 (63%) proposed methodologies under C/D definition with ten estimation methods, three (19%) estimation methods proposed under I/D definition, only one method (6%) proposed under I/S and another two papers (12%) proposed methodologies on some other longitudinal marker methods.

The following Figure B and Figure C are the frequency charts for the 308 clinical application papers that were found from our review according to their year of publications and top disease areas respectively.

**Figure S1B Year of publication for clinical applications under C/D and I/D definitions**

**Figure S1C Disease area for clinical applications under C/D and I/D definitions**

# References

1. Heagerty PJ, Zheng Y: **Survival model predictive accuracy and ROC curves**. *Biometrics* 2005, **61**(1):92-105.

2. Saha-Chaudhuri P, Heagerty PJ: **Non-parametric estimation of a time-dependent predictive accuracy curve**. *Biostatistics* 2013, **14**(1):42-59.

3. Shen W, Ning J, Yuan Y: **A direct method to evaluate the time‐dependent predictive accuracy for biomarkers**. *Biometrics* 2015, **71**(2):439-449.

4. Blanche P, Dartigues JF, Jacqmin-Gadda H: **Review and comparison of ROC curve estimators for a time-dependent outcome with marker-dependent censoring**. *Biometrical Journal* 2013, **55**(5):687-704.

5. Chambless LE, Diao G: **Estimation of time-dependent area under the ROC curve for long-term risk prediction**. *Stat Med* 2006, **25**(20):3474-3486.

6. Heagerty PJ, Lumley T, Pepe MS: **Time-dependent ROC curves for censored survival data and a diagnostic marker**. *Biometrics* 2000, **56**(2):337-344.

7. Hung H, Chiang CT: **Optimal Composite Markers for Time-Dependent Receiver Operating Characteristic Curves with Censored Survival Data**. *Scandinavian Journal of Statistics* 2010, **37**(4):664-679.

8. Hung H, Chiang CT: **Estimation methods for time-dependent AUC models with survival data**. *CANADIAN JOURNAL OF STATISTICS-REVUE CANADIENNE DE STATISTIQUE* 2010, **38**(1):8-26.

9. Lambert J, Chevret S: **Summary measure of discrimination in survival models based on cumulative/dynamic time-dependent ROC curves**. *Statistical Methods In Medical Research* 2014.

10. Chiang C-T, Hung H: **Non‐parametric estimation for time-dependent AUC**. *Journal of Statistical Planning and Inference* 2010, **140**(5):1162-1174.

11. Viallon V, Latouche A: **Discrimination measures for survival outcomes: connection between the AUC and the predictiveness curve**. *Biometrical Journal* 2011, **53**(2):217-236.

12. Zheng Y, Heagerty PJ: **Prospective accuracy for longitudinal markers**. *Biometrics* 2007, **63**(2):332-341.

13. Cai T, Pepe MS, Zheng Y, Lumley T, Jenny NS: **The sensitivity and specificity of markers for event times**. *Biostatistics* 2006, **7**(2):182-197.

14. Etzioni R, Pepe M, Longton G, Hu C, Goodman G: **Incorporating the time dimension in receiver operating characteristic curves: a case study of prostate cancer**. *Medical Decision Making* 1999, **19**(3):242-251.

15. Zheng Y, Heagerty PJ: **Semiparametric estimation of time‐dependent ROC curves for longitudinal marker data**. *Biostatistics* 2004, **5**(4):615-632.

16. Arnold FW, Brock GN, Peyrani P, Rodríguez EL, Díaz AA, Rossi P, Ramirez JA: **Predictive accuracy of the pneumonia severity index vs CRB-65 for time to clinical stability: results from the Community-Acquired Pneumonia Organization (CAPO) International Cohort Study**. *Respiratory medicine* 2010, **104**(11):1736-1743.

17. Shi Y-Y, Li Z-Y, Zhao M-H, Chen M: **The CD4 lymphocyte count is a better predictor of overall infection than the total lymphocyte count in ANCA-associated vasculitis under a corticosteroid and cyclophosphamide regimen: A retrospective cohort**. *Medicine* 2015, **94**(18).

18. Van Nies J, Tsonaka R, Gaujoux-Viala C, Fautrel B, van der Helm-van Mil A: **Evaluating relationships between symptom duration and persistence of rheumatoid arthritis: does a window of opportunity exist? Results on the Leiden Early Arthritis Clinic and ESPOIR cohorts**. *Annals of the rheumatic diseases* 2015, **74**(5):806-812.

19. Bøvelstad HM, Borgan Ø: **Assessment of evaluation criteria for survival prediction from genomic data**. *Biometrical Journal* 2011, **53**(2):202-216.

20. Tapak L, Saidijam M, Sadeghifar M, Poorolajal J, Mahjub H: **Competing risks data analysis with high-dimensional covariates: an application in bladder cancer**. *Genomics, proteomics & bioinformatics* 2015, **13**(3):169-176.

21. Dietrich D, Krispin M, Dietrich J, Fassbender A, Lewin J, Harbeck N, Schmitt M, Eppenberger-Castori S, Vuaroqueaux V, Spyratos F: **CDO1 promoter methylation is a biomarker for outcome prediction of anthracycline treated, estrogen receptor-positive, lymph node-positive breast cancer patients**. *BMC cancer* 2010, **10**(1):1.

22. Wang L, Yao L, Zheng Y-Z, Xu Q, Liu X-P, Hu X, Wang P, Shao Z-M: **Expression of autophagy-related proteins ATG5 and FIP200 predicts favorable disease-free survival in patients with breast cancer**. *Biochemical and biophysical research communications* 2015, **458**(4):816-822.

23. Yasrebi H, Sperisen P, Praz V, Bucher P: **Can survival prediction be improved by merging gene expression data sets?** *PloS one* 2009, **4**(10):e7431.

24. Yue Y, Cui X, Bose S, Audeh W, Zhang X, Fraass B: **Stratifying triple-negative breast cancer prognosis using 18F-FDG-PET/CT imaging**. *Breast cancer research and treatment* 2015, **153**(3):607-616.

25. Cecchini RS, Costantino JP, Cauley JA, Cronin WM, Wickerham DL, Bandos H, Weissfeld JL, Wolmark N: **Baseline mammographic breast density and the risk of invasive breast cancer in postmenopausal women participating in the NSABP study of tamoxifen and raloxifene (STAR)**. *Cancer Prevention Research* 2012, **5**(11):1321-1329.

26. Irie Y, Katakami N, Kaneto H, Takahara M, Nishio M, Kasami R, Sakamoto Ky, Umayahara Y, Sumitsuji S, Ueda Y: **The utility of ultrasonic tissue characterization of carotid plaque in the prediction of cardiovascular events in diabetic patients**. *Atherosclerosis* 2013, **230**(2):399-405.

27. Katakami N, Osonoi T, Takahara M, Saitou M, Matsuoka T-a, Yamasaki Y, Shimomura I: **Clinical utility of brachial-ankle pulse wave velocity in the prediction of cardiovascular events in diabetic patients**. *Cardiovascular diabetology* 2014, **13**(1):1.

28. Liu J, Yang HI, Lee MH, Batrla‐Utermann R, Jen CL, Lu SN, Wang LY, You SL, Hsiao CK, Chen CJ: **Distinct seromarkers predict different milestones of chronic hepatitis B progression**. *Hepatology* 2014, **60**(1):77-86.

29. Adams H, Tzankov A, Lugli A, Zlobec I: **New time-dependent approach to analyse the prognostic significance of immunohistochemical biomarkers in colon cancer and diffuse large B-cell lymphoma**. *Journal of clinical pathology* 2009, **62**(11):986-997.

30. Hyun SH, Choi JY, Shim YM, Kim K, Lee SJ, Cho YS, Lee JY, Lee K-H, Kim B-T: **Prognostic value of metabolic tumor volume measured by 18F-fluorodeoxyglucose positron emission tomography in patients with esophageal carcinoma**. *Annals of surgical oncology* 2010, **17**(1):115-122.

31. Wang C-Y, Lee T-F, Fang C-H, Chou J-H: **Fuzzy logic-based prognostic score for outcome prediction in esophageal cancer**. *IEEE Transactions on Information Technology in Biomedicine* 2012, **16**(6):1224-1230.

32. Cao S-M, Liu Z, Jia W-H, Huang Q-H, Liu Q, Guo X, Huang T-B, Ye W, Hong M-H: **Fluctuations of epstein-barr virus serological antibodies and risk for nasopharyngeal carcinoma: a prospective screening study with a 20-year follow-up**. *PloS one* 2011, **6**(4):e19100.

33. Moon SH, Choi JY, Lee HJ, Son Y-I, Baek C-H, Ahn YC, Ahn M-J, Park K, Kim B-T: **Prognostic value of volume-based positron emission tomography/computed tomography in patients with nasopharyngeal carcinoma treated with concurrent chemoradiotherapy**. *Clinical and experimental otorhinolaryngology* 2015, **8**(2):142-148.

34. Zhang KW, French B, Khan AM, Plappert T, Fang JC, Sweitzer NK, Borlaug BA, Chirinos JA, Sutton MSJ, Cappola TP: **Strain improves risk prediction beyond ejection fraction in chronic systolic heart failure**. *Journal of the American Heart Association* 2014, **3**(1):e000550.

35. Weidmann ZM, Breidthardt T, Twerenbold R, Züsli C, Nowak A, von Eckardstein A, Erne P, Rentsch K, de Oliveira MT, Gualandro D: **Prediction of mortality using quantification of renal function in acute heart failure**. *International journal of cardiology* 2015, **201**:650-657.

36. Simon RM, Subramanian J, Li M-C, Menezes S: **Using cross-validation to evaluate predictive accuracy of survival risk classifiers based on high-dimensional data**. *Briefings in bioinformatics* 2011, **12**(3):203-214.

37. Philosophe B, Malat GE, Soundararajan S, Barth RN, Manitpisikul W, Wilson NS, Ranganna K, Drachenberg CB, Papadimitriou JC, Neuman BP: **Validation of the Maryland Aggregate Pathology Index (MAPI), a pre‐implantation scoring system that predicts graft outcome**. *Clinical transplantation* 2014, **28**(8):897-905.

38. Pipili C, Ioannidou S, Tripodaki E-S, Parisi M, Douka E, Vasileiadis I, Joannidis M, Nanas S: **Prediction of the renal replacement therapy requirement in mechanically ventilated critically ill patients by combining biomarkers for glomerular filtration and tubular damage**. *Journal of critical care* 2014, **29**(4):692. e697-692. e613.

39. Sentís A, Kers J, Yapici U, Claessen N, Roelofs JJ, Bemelman FJ, ten Berge IJ, Florquin S: **The prognostic significance of glomerular infiltrating leukocytes during acute renal allograft rejection**. *Transplant immunology* 2015, **33**(3):168-175.

40. Zhao Y, Zhu L, Zhou T, Zhang Q, Shi S, Liu L, Lv J, Zhang H: **Urinary CXCL1: a novel predictor of IgA nephropathy progression**. *PloS one* 2015, **10**(3):e0119033.

41. Kim CJ, Kim HJ, Park JH, Park DI, Cho YK, Sohn CI, Jeon WK, Kim BI, Kim MJ: **Radiologic response to transcatheter hepatic arterial chemoembolization and clinical outcomes in patients with hepatocellular carcinoma**. *Liver International* 2014, **34**(2):305-312.

42. Li X, Han Z, Cheng Z, Yu J, Yu X, Liang P: **Clinical significance of preoperative platelet-to-lymphocyte ratio in recurrent hepatocellular carcinoma after thermal ablation: A retrospective analysis**. *International Journal of Hyperthermia* 2015, **31**(7):758-763.

43. Li X, Han Z, Cheng Z, Yu J, Liu S, Yu X, Liang P: **Preoperative neutrophil-to-lymphocyte ratio is a predictor of recurrence following thermal ablation for recurrent hepatocellular carcinoma: a retrospective analysis**. *PloS one* 2014, **9**(10):e110546.

44. Kim BK, Han K-H, Park JY, Kim JK, Paik YH, Lee KS, Chon CY, Ahn SH: **Risk assessment of esophageal variceal bleeding in B-viral liver cirrhosis by a liver stiffness measurement-based model**. *The American journal of gastroenterology* 2011, **106**(9):1654-1662.

45. Iio E, Ocho M, Togayachi A, Nojima M, Kuno A, Ikehara Y, Hasegawa I, Yatsuhashi H, Yamasaki K, Shimada N: **A novel glycobiomarker, Wisteria floribunda agglutinin macrophage colony‐stimulating factor receptor, for predicting carcinogenesis of liver cirrhosis**. *International Journal of Cancer* 2016, **138**(6):1462-1471.

46. Kim H-D, Shim JH, Kim G-A, Shin YM, Yu E, Lee S-G, Lee D, Kim KM, Lim Y-S, Lee HC: **Optimal methods for measuring eligibility for liver transplant in hepatocellular carcinoma patients undergoing transarterial chemoembolization**. *Journal of hepatology* 2015, **62**(5):1076-1084.

47. Dai J, Hu Z, Dong J, Xu L, Pan S, Jiang Y, Jin G, Chen Y, Shen H: **Host immune gene polymorphisms were associated with the prognosis of non‐small‐cell lung cancer in Chinese**. *International Journal of Cancer* 2012, **130**(3):671-676.

48. Cao S, Wang C, Huang X, Dai J, Hu L, Liu Y, Chen J, Ma H, Jin G, Hu Z: **Prognostic assessment of apoptotic gene polymorphisms in non-small cell lung cancer in Chinese**. *J Biomed Res* 2013, **27**(3):231-238.

49. Hyun SH, Ahn HK, Kim H, Ahn M-J, Park K, Ahn YC, Kim J, Shim YM, Choi JY: **Volume-based assessment by 18F-FDG PET/CT predicts survival in patients with stage III non-small-cell lung cancer**. *European journal of nuclear medicine and molecular imaging* 2014, **41**(1):50-58.

50. Hyun SH, Choi JY, Kim K, Kim J, Shim YM, Um S-W, Kim H, Lee K-H, Kim B-T: **Volume-Based Parameters of 18F-Fluorodeoxyglucose Positron Emission Tomography/Computed Tomography Improve Outcome Prediction in Early-Stage Non–Small Cell Lung Cancer After Surgical Resection**. *Annals of surgery* 2013, **257**(2):364-370.

51. Lu Y, Wang L, Liu P, Yang P, You M: **Gene-expression signature predicts postoperative recurrence in stage I non-small cell lung cancer patients**. *PLoS One* 2012, **7**(1):e30880.

52. Yan H, Wang R, Zhao F, Zhu K, Jiang S, Zhao W, Feng R: **Measurement of tumor volume by PET to evaluate prognosis in patients with advanced non-small cell lung cancer treated by non-surgical therapy**. *Acta Radiologica* 2011, **52**(6):646-650.

53. Gries CJ, Rue TC, Heagerty PJ, Edelman JD, Mulligan MS, Goss CH: **Development of a predictive model for long-term survival after lung transplantation and implications for the lung allocation score**. *The Journal of Heart and Lung Transplantation* 2010, **29**(7):731-738.

54. Tse LA, Dai J, Chen M, Liu Y, Zhang H, Wong TW, Leung CC, Kromhout H, Meijer E, Liu S: **Prediction models and risk assessment for silicosis using a retrospective cohort study among workers exposed to silica in China**. *Scientific reports* 2015, **5**.

55. Gattolliat CH, Le Teuff G, Combaret V, Mussard E, Valteau‐Couanet D, Busson P, Bénard J, Douc‐Rasy S: **Expression of two parental imprinted miRNAs improves the risk stratification of neuroblastoma patients**. *Cancer medicine* 2014, **3**(4):998-1009.

56. Hamidi O, Tapak L, Jafarzadeh Kohneloo A, Sadeghifar M: **High-Dimensional Additive Hazards Regression for Oral Squamous Cell Carcinoma Using Microarray Data: A Comparative Study**. *BioMed research international* 2014, **2014**.

57. Oikonomopoulou K, Li L, Zheng Y, Simon I, Wolfert R, Valik D, Nekulova M, Simickova M, Frgala T, Diamandis E: **Prediction of ovarian cancer prognosis and response to chemotherapy by a serum-based multiparametric biomarker panel**. *British journal of cancer* 2008, **99**(7):1103-1113.

58. Sohn I, Sung CO: **Predictive modeling using a somatic mutational profile in ovarian high grade serous carcinoma**. *PLoS One* 2013, **8**(1):e54089.

59. Fall K, Garmo H, Andrén O, Bill-Axelson A, Adolfsson J, Adami H-O, Johansson J-E, Holmberg L: **Prostate-specific antigen levels as a predictor of lethal prostate cancer**. *Journal of the National Cancer Institute* 2007, **99**(7):526-532.

60. Ross AE, Johnson MH, Yousefi K, Davicioni E, Netto GJ, Marchionni L, Fedor HL, Glavaris S, Choeurng V, Buerki C: **Tissue-based genomics augments post-prostatectomy risk stratification in a natural history cohort of intermediate-and high-risk men**. *European urology* 2016, **69**(1):157-165.

61. Ross AE, Feng F, Ghadessi M, Erho N, Crisan A, Buerki C, Sundi D, Mitra A, Vergara I, Thompson D: **A genomic classifier predicting metastatic disease progression in men with biochemical recurrence after prostatectomy**. *Prostate cancer and prostatic diseases* 2014, **17**(1):64-69.

62. Robinson D, Sandblom G, Johansson R, Garmo H, Stattin P, Mommsen S, Varenhorst E, Group SPC: **Prediction of survival of metastatic prostate cancer based on early serial measurements of prostate specific antigen and alkaline phosphatase**. *The Journal of urology* 2008, **179**(1):117-123.

63. Ross AE, Yousefi K, Davicioni E, Ghadessi M, Johnson MH, Sundi D, Tosoian JJ, Han M, Humphreys EB, Partin AW: **Utility of risk models in decision making after radical prostatectomy: lessons from a natural history cohort of intermediate-and high-risk men**. *European urology* 2016, **69**(3):496-504.

64. Tarhini AA, Lin Y, Zahoor H, Shuai Y, Butterfield LH, Ringquist S, Gogas H, Sander C, Lee S, Agarwala SS: **Pro-Inflammatory cytokines predict relapse-free survival after one month of Interferon-α but not observation in intermediate risk melanoma patients**. *PloS one* 2015, **10**(7):e0132745.

65. Zaragoza J, Caille A, Beneton N, Bens G, Christiann F, Maillard H, Machet L: **High neutrophil to lymphocyte ratio measured before starting ipilimumab treatment is associated with reduced overall survival in patients with melanoma**. *British Journal of Dermatology* 2016, **174**(1):146-151.

66. Pradhan TS, Stevens EE, Ablavsky M, Salame G, Lee Y-C, Abulafia O: **FIGO staging for carcinosarcoma: can the revised staging system predict overall survival?** *Gynecologic oncology* 2011, **123**(2):221-224.

67. Yue Y, Astvatsaturyan K, Cui X, Zhang X, Fraass B, Bose S: **Stratification of Prognosis of Triple-Negative Breast Cancer Patients Using Combinatorial Biomarkers**. *PloS one* 2016, **11**(3):e0149661.

68. Shah RV, Truong QA, Gaggin HK, Pfannkuche J, Hartmann O, Januzzi JL: **Mid-regional pro-atrial natriuretic peptide and pro-adrenomedullin testing for the diagnostic and prognostic evaluation of patients with acute dyspnoea**. *European heart journal* 2012, **33**(17):2197-2205.

69. Sanford R, Chavez-MacGregor M: **Time to initiation of adjuvant chemotherapy in patients with rapidly proliferating early breast cancer**. *Breast Diseases: a YB Quarterly* 2016, **27**(3):221-222.

70. Zhou D, Zhang Y, Xu L, Zhou Z, Huang J, Chen M: **A monocyte/granulocyte to lymphocyte ratio predicts survival in patients with hepatocellular carcinoma**. *Scientific reports* 2015, **5**.

71. Lin Y, Lin W-Y, Kao C-H, Yen K-Y, Chen S-W, Yeh J-J: **Prognostic value of preoperative metabolic tumor volumes on PET-CT in predicting disease-free survival of patients with stage I non-small cell lung cancer**. *Anticancer research* 2012, **32**(11):5087-5091.

72. Nikiphorou E, Kautiainen H, Hannonen P, Asikainen J, Kokko A, Rannio T, Sokka T: **Clinical effectiveness of CT-P13 (Infliximab biosimilar) used as a switch from Remicade (infliximab) in patients with established rheumatic disease. Report of clinical experience based on prospective observational data**. *Expert opinion on biological therapy* 2015, **15**(12):1677-1683.

73. Sørensen M, Gerds T, Hindsø K, Petersen M: **Prediction of survival after surgery due to skeletal metastases in the extremities**. *Bone Joint J* 2016, **98**(2):271-277.

74. Ludka O, Štípal R, Šenkyříková M, Musil V, Trna J, Pozdíšek Z, Jarkovský J, Dušek L, Špinar J: **The importance of admission and discharge BNP assessment in patients hospitalized for acutely decompensated chronic systolic heart failure**. In: *EUROPEAN JOURNAL OF HEART FAILURE: 2014*: WILEY-BLACKWELL 111 RIVER ST, HOBOKEN 07030-5774, NJ USA; 2014: 231-232.

75. Wreesmann VB, Katabi N, Palmer FL, Montero PH, Migliacci JC, Gönen M, Carlson D, Ganly I, Shah JP, Ghossein R: **Influence of extracapsular nodal spread extent on prognosis of oral squamous cell carcinoma**. *Head & neck* 2015.

76. Berg KD, Røder MA, Thomsen FB, Vainer B, Gerds TA, Brasso K, Iversen P: **The predictive value of ERG protein expression for development of castration‐resistant prostate cancer in hormone‐naïve advanced prostate cancer treated with primary androgen deprivation therapy**. *The Prostate* 2015, **75**(14):1499-1509.

77. Hyun D, Shin SW, Cho SK, Park KB, Park HS, Choo SW, Do YS, Choo I-w, Shin J-W, Lim S-J: **Efficacy of RECIST and mRECIST criteria as prognostic factors in patients undergoing repeated iodized oil chemoembolization of intermediate stage hepatocellular carcinoma**. *Acta Radiologica* 2014:0284185114560937.

78. Demissei BG, Valente MA, Cleland JG, O'Connor CM, Metra M, Ponikowski P, Teerlink JR, Cotter G, Davison B, Givertz MM: **Optimizing clinical use of biomarkers in high‐risk acute heart failure patients**. *European journal of heart failure* 2015.

79. Kim BK, Kim SU, Park JY, Kim DY, Ahn SH, Park MS, Kim EH, Seong J, Lee DY, Han KH: **Applicability of BCLC stage for prognostic stratification in comparison with other staging systems: single centre experience from long‐term clinical outcomes of 1717 treatment‐naïve patients with hepatocellular carcinoma**. *Liver International* 2012, **32**(7):1120-1127.

80. Derby CA, Burns LC, Wang C, Katz MJ, Zimmerman ME, L’Italien G, Guo Z, Berman RM, Lipton RB: **Screening for predementia AD time-dependent operating characteristics of episodic memory tests**. *Neurology* 2013, **80**(14):1307-1314.

81. Kim MN, Kim SU, Park JY, Han K-H, Chon CY, Ahn SH: **Risk assessment of liver-related events using transient elastography in patients with chronic hepatitis B receiving entecavir**. *Journal of clinical gastroenterology* 2014, **48**(3):272-278.

82. Sigirli D, Ercan I, Jin Y, Paksoy E, Tolunay S, Tasdelen I: **EVALUATING CUT-OFF VALUES WITH TIME-DEPENDENT ROC CURVES FOR Ki67**. *Pakistan Journal of Statistics* 2010, **26**(4).

83. Huang Y, Murakami T, Sano F, Kondo K, Nakaigawa N, Kishida T, Kubota Y, Nagashima Y, Yao M: **Expression of aquaporin 1 in primary renal tumors: a prognostic indicator for clear-cell renal cell carcinoma**. *European urology* 2009, **56**(4):690-699.

84. Kodar K, Izotova J, Klaamas K, Sergeyev B, Järvekülg L, Kurtenkov O: **Aberrant glycosylation of the anti-Thomsen-Friedenreich glycotope immunoglobulin G in gastric cancer patients**. *World J Gastroenterol* 2013, **19**(23):3573–3582.

85. Zhang H, Su Y, Xu F, Kong J, Yu H, Qian B: **Circulating microRNAs in relation to EGFR status and survival of lung adenocarcinoma in female non-smokers**. *PLoS One* 2013, **8**(11):e81408.

86. Rincón D, Lo Iacono O, Tejedor M, Hernando A, Ripoll C, Catalina M-V, Salcedo M, Matilla A, Senosiain M, Clemente G: **Prognostic value of hepatic venous pressure gradient in patients with compensated chronic hepatitis C-related cirrhosis**. *Scandinavian journal of gastroenterology* 2013, **48**(4):487-495.

87. Oh HJ, Lee MJ, Lee HS, Park JT, Han SH, Yoo T-H, Kim Y-L, Kim YS, Yang CW, Kim N-H: **NT-proBNP: is it a more significant risk factor for mortality than troponin T in incident hemodialysis patients?** *Medicine* 2014, **93**(27).

88. Oh HJ, Lee MJ, Kwon YE, Park KS, Park JT, Han SH, Yoo T-H, Kim Y-L, Kim YS, Yang CW: **Which Biomarker is the Best for Predicting Mortality in Incident Peritoneal Dialysis Patients: NT-ProBNP, Cardiac TnT, or hsCRP?: A Prospective Observational Study**. *Medicine* 2015, **94**(44):e1636.

89. Molica S, Giannarelli D, Gentile M, Cutrona G, Di Renzo N, Di Raimondo F, Neri A, Federico M, Ferrarini M, Morabito F: **The utility of two prognostic models for predicting time to first treatment in early chronic lymphocytic leukemia patients: Results of a comparative analysis**. *Leukemia research* 2013, **37**(8):943-947.

90. Mima K, Beppu T, Ishiko T, Chikamoto A, Nakagawa S, Hayashi H, Watanabe M, Sakamaki K, Baba H: **Preoperative serum hyaluronic acid level as a prognostic factor in patients undergoing hepatic resection for hepatocellular carcinoma**. *British Journal of Surgery* 2014, **101**(3):269-276.

91. Ma S, Song X: **Ranking prognosis markers in cancer genomic studies**. *Briefings in bioinformatics* 2011, **12**(1):33-40.

92. López-Otero D, Trillo-Nouche R, Gude F, Cid-Álvarez B, Ocaranza-Sanchez R, Álvarez MS, Lear PV, Gonzalez-Juanatey JR: **Pro B-type natriuretic peptide plasma value: a new criterion for the prediction of short-and long-term outcomes after transcatheter aortic valve implantation**. *International journal of cardiology* 2013, **168**(2):1264-1268.

93. Kubozono T, Itoh H, Oikawa K, Tajima A, Maeda T, Aizawa T, Iinuma H, Tokuda Y, Ohashi Y, Fu LT: **Peak VO2 is more potent than B-type natriuretic peptide as a prognostic parameter in cardiac patients**. *Circulation Journal* 2007, **72**(4):575-581.

94. Jankowitz RC, Cooper K, Erlander MG, Ma X-J, Kesty NC, Li H, Chivukula M, Brufsky A: **Prognostic utility of the breast cancer index and comparison to Adjuvant! Online in a clinical case series of early breast cancer**. *Breast Cancer Research* 2011, **13**(5):1.

95. Guler I, Calaza-Díaz L, Faes C, Cadarso-Suárez C, Giraldez E, Gude F: **Joint Modelling for Longitudinal and Time-to-Event Data: Application to Liver Transplantation Data**. In: *International Conference on Computational Science and Its Applications: 2014*: Springer; 2014: 580-593.

96. George J, Claes P, Vunckx K, Tejpar S, Deroose C, Nuyts J, Loeckx D, Suetens P: **A textural feature based tumor therapy response prediction model for longitudinal evaluation with PET imaging**. In: *2012 9th IEEE International Symposium on Biomedical Imaging (ISBI): 2012*: IEEE; 2012: 1048-1051.

97. Desmedt C, Giobbie-Hurder A, Neven P, Paridaens R, Christiaens M-R, Smeets A, Lallemand F, Haibe-Kains B, Viale G, Gelber RD: **The Gene expression Grade Index: a potential predictor of relapse for endocrine-treated breast cancer patients in the BIG 1–98 trial**. *BMC medical genomics* 2009, **2**(1):1.

98. Okereke OI, Pantoja-Galicia N, Copeland M, Hyman BT, Wanggaard T, Albert MS, Betensky RA, Blacker D: **The SIST-M: predictive validity of a brief structured clinical dementia rating interview**. *Alzheimer disease and associated disorders* 2012, **26**(3):225.

99. Schmid M, Kestler HA, Potapov S: **On the validity of time-dependent AUC estimators**. *Briefings in bioinformatics* 2013:bbt059.

100. Sartipy U, Goda A, Yuzefpolskaya M, Mancini DM, Lund LH: **Utility of the Seattle Heart Failure Model in patients with cardiac resynchronization therapy and implantable cardioverter defibrillator referred for heart transplantation**. *American heart journal* 2014, **168**(3):325-331.

101. Kuang TM, Zhang C, Zangwill LM, Weinreb RN, Medeiros FA: **Estimating lead time gained by optical coherence tomography in detecting glaucoma before development of visual field defects**. *Ophthalmology* 2015, **122**(10):2002-2009.

102. Abdi ZD, Essig M, Rizopoulos D, Le Meur Y, Prémaud A, Woillard J-B, Rerolle J-P, Marquet P, Rousseau A: **Impact of longitudinal exposure to mycophenolic acid on acute rejection in renal-transplant recipients using a joint modeling approach**. *Pharmacological research* 2013, **72**:52-60.

103. Kim HL, Halabi S, Li P, Mayhew G, Simko J, Nixon AB, Small EJ, Rini B, Morris MJ, Taplin M-E: **A molecular model for predicting overall survival in patients with metastatic clear cell renal carcinoma: results from CALGB 90206 (Alliance)**. *EBioMedicine* 2015, **2**(11):1814-1820.

104. Gerke TA, Martin NE, Ding Z, Nuttall EJ, Stack EC, Giovannucci E, Lis RT, Stampfer MJ, Kantoff PW, Parmigiani G: **Evaluating a 4‐marker signature of aggressive prostate cancer using time‐dependent AUC**. *The Prostate* 2015, **75**(16):1926-1933.

105. Pagano MJ, Whalen MJ, Paulucci DJ, Reddy BN, Matulay JT, Rothberg M, Scarberry K, Patel T, Shapiro EY, RoyChoudhury A: **Predictors of biochemical recurrence in pT3b prostate cancer after radical prostatectomy without adjuvant radiotherapy**. *The Prostate* 2016, **76**(2):226-234.

106. Chen H-Y, Chiu Y-L, Chuang Y-F, Hsu S-P, Pai M-F, Yang J-Y, Peng Y-S: **Visceral adiposity index and risks of cardiovascular events and mortality in prevalent hemodialysis patients**. *Cardiovascular diabetology* 2014, **13**(1):1.

107. Zhou M, Zhao H, Wang Z, Cheng L, Yang L, Shi H, Yang H, Sun J: **Identification and validation of potential prognostic lncRNA biomarkers for predicting survival in patients with multiple myeloma**. *Journal of Experimental & Clinical Cancer Research* 2015, **34**(1):1.

108. Brant LJ, Ferrucci L, Sheng SL, Concin H, Zonderman AB, Kelleher CC, Longo DL, Ulmer H, Strasak AM: **Gender differences in the accuracy of time-dependent blood pressure indices for predicting coronary heart disease: A random-effects modeling approach**. *Gender medicine* 2010, **7**(6):616-627.

109. Hintz SR, Bann CM, Ambalavanan N, Cotten CM, Das A, Higgins RD: **Predicting time to hospital discharge for extremely preterm infants**. *Pediatrics* 2010, **125**(1):e146-e154.

110. Plischke M, Neuhold S, Kohl M, Heinze G, Sunder‐Plassmann G, Pacher R, Hülsmann M: **Renal function in heart failure: a disparity between estimating function and predicting mortality risk**. *European journal of heart failure* 2013, **15**(7):763-770.

111. Alansari SE, Croal BL: **Diagnostic value of heart fatty acid binding protein and myoglobin in patients admitted with chest pain**. *Annals of clinical biochemistry* 2004, **41**(5):391-396.

112. Aoyama K, Fushimi Y, Okada T, Miyasaki A, Taki H, Shibamoto K, Togashi K: **Detection of symptomatic vasospasm after subarachnoid haemorrhage: initial findings from single time-point and serial measurements with arterial spin labelling**. *European radiology* 2012, **22**(11):2382-2391.

113. Ashton M, Gordi T, Hai TN, Van Huong N, Sy ND, Nieu NT, Huong DX, Johansson M, Công LD: **Artemisinin pharmacokinetics in healthy adults after 250, 500 and 1000 mg single oral doses**. *Biopharmaceutics & drug disposition* 1998, **19**(4):245-250.

114. Ashton M, Hai TN, Sy ND, Huong DX, Van Huong N, Nieu NT: **Artemisinin pharmacokinetics is time-dependent during repeated oral administration in healthy male adults**. *Drug metabolism and disposition* 1998, **26**(1):25-27.

115. Barbier AJ, Hilhorst M, Van Vliet A, Snyder P, Palfreyman MG, Gawryl M, Dgetluck N, Massaro M, Tiessen R, Timmerman W: **Pharmacodynamics, Pharmacokinetics, Safety, and Tolerability of Encenicline, a Selective α 7 Nicotinic Receptor Partial Agonist, in Single Ascending-dose and Bioavailability Studies**. *Clinical therapeutics* 2015, **37**(2):311-324.

116. Barger A, Fuhst C, Wiedemann B: **Pharmacological indices in antibiotic therapy**. *Journal of Antimicrobial Chemotherapy* 2003, **52**(6):893-898.

117. Bi GD, Jun L, Nekka F: **Antimicrobial breakpoint estimation accounting for variability in pharmacokinetics**. *Theoretical Biology and Medical Modelling* 2009, **6**(1):1.

118. Bouchard J, Malhotra R, Shah S, Kao Y-T, Vaida F, Gupta A, Berg DT, Grinnell BW, Stofan B, Tolwani AJ: **Levels of protein C and soluble thrombomodulin in critically ill patients with acute kidney injury: a multicenter prospective observational study**. *PloS one* 2015, **10**(3):e0120770.

119. Brashier B, Dhembare P, Jantikar A, Mahadik P, Gokhale P, Gogtay J, Salvi S: **Tiotropium administered by a pressurized metered dose inhaler (pMDI) and spacer produces a similar bronchodilator response as that administered by a Rotahaler® in adult subjects with stable moderate-to-severe COPD**. *Respiratory medicine* 2007, **101**(12):2464-2471.

120. Briceno J, Sanchez-Hidalgo J, Naranjo A, Ciria R, Pozo J, Luque A, de la Mata M, Rufián S, López-Cillero P: **Model for end-stage liver disease can predict very early outcome after liver transplantation**. In: *Transplantation proceedings: 2008*: Elsevier; 2008: 2952-2954.

121. Brown SL, Amand MDS, Zamble E: **The dynamic prediction of criminal recidivism: A three-wave prospective study**. *Law and human behavior* 2009, **33**(1):25-45.

122. Buijk S, Gyssens I, Mouton J, Metselaar H, Groenland T, Verbrugh H, Bruining H: **Perioperative pharmacokinetics of cefotaxime in serum and bile during continuous and intermittent infusion in liver transplant patients**. *Journal of Antimicrobial Chemotherapy* 2004, **54**(1):199-205.

123. Calvert H, Twelves C, Ranson M, Plummer R, Fettner S, Pantze M, Ling J, Hamilton M, Lum BL, Rakhit A: **Effect of erlotinib on CYP3A activity, evaluated in vitro and by dual probes in patients with cancer**. *Anti-cancer drugs* 2014, **25**(7):832-840.

124. Cannady EA, Suico JG, Wang MD, Friedrich S, Rehmel JR, Nicholls SJ, Krueger KA: **CYP‐mediated drug–drug interactions with evacetrapib, an investigational CETP inhibitor: in vitro prediction and clinical outcome**. *British journal of clinical pharmacology* 2015, **80**(6):1388-1398.

125. Carsenti-Etesse H, Farinotti R, Durant J, Roger P, De Salvador F, Bernard E, Rouveix B, Dellamonica P: **Pharmacokinetic parameters and killing rates in serum of volunteers receiving amoxicillin, cefadroxil or cefixime alone or associated with niflumic acid or paracetamol**. *European journal of drug metabolism and pharmacokinetics* 1998, **23**(3):357-366.

126. Cha Y-J, Lim KS, Park M-K, Schneider S, Bray B, Kang M-C, Chung J-Y, Yoon SH, Cho J-Y, Yu K-S: **Pharmacokinetics and tolerability of the new second-generation nonnucleoside reverse-transcriptase inhibitor KM-023 in healthy subjects**. *Drug design, development and therapy* 2014, **8**:1613.

127. Chang S-Y, Fancher R, Zhang H, Gan J: **Mechanism-based inhibition of human cytochrome P4503A4 by domperidone**. *Xenobiotica* 2010, **40**(2):138-145.

128. Chiu WA, White P: **Steady‐state solutions to PBPK models and their applications to risk assessment I: route‐to‐route extrapolation of volatile chemicals**. *Risk Analysis* 2006, **26**(3):769-780.

129. Coelho LM, Salluh JI, Soares M, Bozza FA, Verdeal JR, Castro-Faria-Neto HC, e Silva JRL, Bozza PT, Póvoa P: **Patterns of c-reactive protein RATIO response in severe community-acquired pneumonia: a cohort study**. *Critical Care* 2012, **16**(2):1.

130. Craig WA: **Choosing an antibiotic on the basis of pharmacodynamics**. *Ear, nose & throat journal* 1998, **77**(6):7.

131. CZEJKA M, SCHUELLER J, HAUER K, OSTERMANN E: **Pharmacokinetics and metabolism of irinotecan combined with capecitabine in patients with advanced colorectal cancer**. *Anticancer research* 2005, **25**(4):2985-2990.

132. de Geus HR, Fortrie G, Betjes MG, van Schaik RH, Groeneveld AJ: **Time of injury affects urinary biomarker predictive values for acute kidney injury in critically ill, non-septic patients**. *BMC nephrology* 2013, **14**(1):1.

133. de Lange EC, Hesselink MB, Danhof M, de Boer AG, Breimer DD: **The use of intracerebral microdialysis to determine changes in blood-brain barrier transport characteristics**. *Pharmaceutical research* 1995, **12**(1):129-133.

134. Delbaldo C, Chatelut E, Ré M, Deroussent A, Séronie-Vivien S, Jambu A, Berthaud P, Le Cesne A, Blay J-Y, Vassal G: **Pharmacokinetic-pharmacodynamic relationships of imatinib and its main metabolite in patients with advanced gastrointestinal stromal tumors**. *Clinical Cancer Research* 2006, **12**(20):6073-6078.

135. Dhuria S, Einolf H, Mangold J, Sen S, Gu H, Wang L, Cameron S: **Time‐Dependent Inhibition and Induction of Human Cytochrome P4503A4/5 by an Oral IAP Antagonist, LCL161, In Vitro and In Vivo in Healthy Subjects**. *The Journal of Clinical Pharmacology* 2013, **53**(6):642-653.

136. Donnenberg V, Wilson J, Burckart G, Zeevi A, Iacono A, Donnenberg A: **Measurement of basal, substrate induced and total P‐glycoprotein activity in bronchoalveolar lavage T‐cell subsets**. *Cytometry Part A* 2004, **57**(2):75-85.

137. Dowell JA, Goldstein BP, Buckwalter M, Stogniew M, Damle B: **Pharmacokinetic‐Pharmacodynamic Modeling of Dalbavancin, a Novel Glycopeptide Antibiotic**. *The Journal of Clinical Pharmacology* 2008, **48**(9):1063-1068.

138. Fang ZZ, Zhang YY, Ge GB, Huo H, Liang SC, Yang L: **Time‐dependent inhibition (TDI) of CYP3A4 and CYP2C9 by noscapine potentially explains clinical noscapine–warfarin interaction**. *British journal of clinical pharmacology* 2010, **69**(2):193-199.

139. Felipe CR, Silva Jr HT, Machado PGP, Garcia R, da Silva Moreira SR, Pestana JOM: **Time-dependent changes in cyclosporine exposure: implications for achieving target concentrations**. *Transplant International* 2003, **16**(7):494-503.

140. Fonseca I, Reguengo H, Almeida M, Dias L, Martins LS, Pedroso S, Santos J, Lobato L, Henriques AC, Mendonça D: **Oxidative stress in kidney transplantation: malondialdehyde is an early predictive marker of graft dysfunction**. *Transplantation* 2014, **97**(10):1058-1065.

141. Frassetto L, Floren L, Barin B, Browne M, Wolfe A, Roland M, Stock P, Carlson L, Christians U, Benet L: **Changes in clearance, volume and bioavailability of immunosuppressants when given with HAART in HIV‐1 infected liver and kidney transplant recipients**. *Biopharmaceutics & drug disposition* 2013, **34**(8):442-451.

142. Galetin A, Burt H, Gibbons L, Houston JB: **Prediction of time-dependent CYP3A4 drug-drug interactions: impact of enzyme degradation, parallel elimination pathways, and intestinal inhibition**. *Drug metabolism and disposition* 2006, **34**(1):166-175.

143. Gao L, Zhu Y, Lyu Y, Hao F-L, Zhang P, Wei M-J: **A Pharmacokinetic and Pharmacodynamic Study on Intravenous Cefazedone Sodium in Patients with Community-acquired Pneumonia**. *Chinese medical journal* 2015, **128**(9):1160.

144. Gasparini S, Ferlazzo E, Beghi E, Tripepi G, Labate A, Mumoli L, Leonardi CG, Cianci V, Latella MA, Gambardella A: **Family history and frontal lobe seizures predict long-term remission in newly diagnosed cryptogenic focal epilepsy**. *Epilepsy research* 2013, **107**(1):101-108.

145. Ghimire M, McInnes FJ, Watson DG, Mullen AB, Stevens HN: **In-vitro/in-vivo correlation of pulsatile drug release from press-coated tablet formulations: a pharmacoscintigraphic study in the beagle dog**. *European Journal of Pharmaceutics and Biopharmaceutics* 2007, **67**(2):515-523.

146. Ghio L, Ferraresso M, Vigano S, Ginevri F, Perfumo F, Gianoglio B, Murer L, Zacchello G, Strologo LD, Cardillo M: **Mycophenolate mofetil pharmacokinetic monitoring in pediatric kidney transplant recipients**. In: *Transplantation proceedings: 2005*: Elsevier; 2005: 856-858.

147. Gordi T, Xie R, Huong NV, Huong DX, Karlsson MO, Ashton M: **A semiphysiological pharmacokinetic model for artemisinin in healthy subjects incorporating autoinduction of metabolism and saturable first‐pass hepatic extraction**. *British journal of clinical pharmacology* 2005, **59**(2):189-198.

148. Groll AH, Giri N, Petraitis V, Petraitiene R, Candelario M, Bacher JS, Piscitelli SC, Walsh TJ: **Comparative efficacy and distribution of lipid formulations of amphotericin B in experimental Candida albicans infection of the central nervous system**. *Journal of Infectious Diseases* 2000, **182**(1):274-282.

149. Güney HZ, Hodog'lugil U, Uluog'lu C, Görgün CZ, Ercan ZS, Lu NA, Zengil H: **In Vitro Susceptibility Rhythms. II. Biological-Time-Dependent Differences in Effect of βr and β2-Adrenergic Agonists of Rat Aorta and Influence of Endothelium**. *Chronobiology international* 1998, **15**(2):159-172.

150. Güney HZ, Uluoĝglu C, Hodoĝglugil U, Görgün CZ, Yamanoĝglu TM, Abacioĝglu N, Zengil H: **Biological-time-dependent differences in effect of verapamil on rat aorta and influence of endothelium**. *Chronobiology international* 1999, **16**(6):779-787.

151. Guo J, Zhou D, Li Y, Khanh BH: **Physiologically based pharmacokinetic modeling to predict complex drug–drug interactions: a case study of AZD2327 and its metabolite, competitive and time‐dependent CYP3A inhibitors**. *Biopharmaceutics & drug disposition* 2015, **36**(8):507-519.

152. Hardinger KL, Park JM, Schnitzler MA, Koch MJ, Miller BW, Brennan DC: **Pharmacokinetics of tacrolimus in kidney transplant recipients: twice daily versus once daily dosing**. *American Journal of Transplantation* 2004, **4**(4):621-625.

153. Haroldsen PE, Garovoy MR, Musson DG, Zhou H, Tsuruda L, Hanson B, O’Neill CA: **Genetic variation in aryl N-acetyltransferase results in significant differences in the pharmacokinetic and safety profiles of amifampridine (3, 4-diaminopyridine) phosphate**. *Pharmacology research & perspectives* 2015, **3**(1).

154. Hengzhuang W, Ciofu O, Yang L, Wu H, Song Z, Oliver A, Høiby N: **High β-lactamase levels change the pharmacodynamics of β-lactam antibiotics in Pseudomonas aeruginosa biofilms**. *Antimicrobial agents and chemotherapy* 2013, **57**(1):196-204.

155. Hengzhuang W, Wu H, Ciofu O, Song Z, Høiby N: **In vivo pharmacokinetics/pharmacodynamics of colistin and imipenem in Pseudomonas aeruginosa biofilm infection**. *Antimicrobial agents and chemotherapy* 2012, **56**(5):2683-2690.

156. Hsu A, Granneman GR, Witt G, Locke C, Denissen J, Molla A, Valdes J, Smith J, Erdman K, Lyons N: **Multiple-dose pharmacokinetics of ritonavir in human immunodeficiency virus-infected subjects**. *Antimicrobial agents and chemotherapy* 1997, **41**(5):898-905.

157. Hu C, Tompson D, Magee M, Chen Q, Liu YM, Zhu W, Zhao H, Gross AS, Liu Y: **Single and Multiple Dose Pharmacokinetics, Pharmacodynamics and Safety of the Novel Lipoprotein-Associated Phospholipase A 2 Enzyme Inhibitor Darapladib in Healthy Chinese Subjects: An Open Label Phase-1 Clinical Trial**. *PloS one* 2015, **10**(10):e0139862.

158. Huang H, Huang S, Zhu P, Xi X: **Continuous versus intermittent infusion of cefepime in neurosurgical patients with post-operative intracranial infections**. *International journal of antimicrobial agents* 2014, **43**(1):68-72.

159. Huang O, Lu X, Xu X, Shi Y: **Fibrin-Sealant-Delivered Cisplatin Chemotherapy Versus Cisplatin Hyperthermic Intraperitoneal Perfusion Chemotherapy for Locally Advanced Gastric Cancer Without Peritoneal Metastases: A Randomized Phase-II Clinical Trial with a 40-Month Follow-up**. *Cell biochemistry and biophysics* 2015, **71**(2):1171-1180.

160. Huang Y, Li T, Eatherton A, Mitchell WL, Rong N, Ye L, Yang X-J, Jin S, Ding Y, Zhang J: **Orally bioavailable and brain-penetrant pyridazine and pyridine-derived γ-secretase modulators reduced amyloidogenic Aβ peptides in vivo**. *Neuropharmacology* 2013, **70**:278-286.

161. Inui N, Kato T, Uchida S, Chida K, Takeuchi K, Kimura T, Watanabe H: **Novel patch for transdermal administration of morphine**. *Journal of pain and symptom management* 2012, **44**(4):479-485.

162. Jacobs M: **Optimisation of antimicrobial therapy using pharmacokinetic and pharmacodynamic parameters**. *Clinical microbiology and Infection* 2001, **7**(11):589-596.

163. Jayasagar G, Kumar MK, Chandrasekhar K, Rao CM, Rao YM: **Effect of cephalexin on the pharmacokinetics of metformin in healthy human volunteers**. In*.*; 2002.

164. Johne A, Brockmöller J, Bauer S, Maurer A, Langheinrich M, Roots I: **Pharmacokinetic interaction of digoxin with an herbal extract from St John's wort (Hypericum perforatum)**. *Clinical Pharmacology & Therapeutics* 1999, **66**(4):338-345.

165. Jung HY, Kang HE, Choi YH, Kim SH, Lee MG: **Time‐dependent effects of Klebsiella pneumoniae endotoxin on the pharmacokinetics of chlorzoxazone and its main metabolite, 6‐hydroxychlorzoxazone, in rats: restoration of the parameters in 96 hour in KPLPS rats to control levels**. *Biopharmaceutics & drug disposition* 2009, **30**(8):485-493.

166. Jusko WJ: **Pharmacokinetics and receptor-mediated pharmacodynamics of corticosteroids**. *Toxicology* 1995, **102**(1):189-196.

167. Kagan ML, West AL, Zante C, Calder PC: **Acute appearance of fatty acids in human plasma–a comparative study between polar-lipid rich oil from the microalgae Nannochloropsis oculata and krill oil in healthy young males**. *Lipids in health and disease* 2013, **12**(1):1.

168. Kakuda T, Sekar V, Vis P, Coate B, Ryan R, Anderson D, De La Rosa G, Mrus J: **Pharmacokinetics and Pharmacodynamics of Darunavir and Etravirine in HIV-1–Infected, Treatment-Experienced Patients in the Gender, Race, and Clinical Experience (GRACE) Trial**. *AIDS research and treatment* 2012, **2012**.

169. Kanat M, Mari A, Norton L, Winnier D, DeFronzo RA, Jenkinson C, Abdul-Ghani MA: **Distinct β-cell defects in impaired fasting glucose and impaired glucose tolerance**. *Diabetes* 2012, **61**(2):447-453.

170. Kanda M, Fujii T, Takami H, Suenaga M, Inokawa Y, Yamada S, Kobayashi D, Tanaka C, Sugimoto H, Koike M: **Novel diagnostics for aggravating pancreatic fistulas at the acute phase after pancreatectomy**. *World journal of gastroenterology: WJG* 2014, **20**(26):8535.

171. Karwa R, Shashank A, Rambhau D, Gopinath D, Ravi D: **Time-Dependent Pharmacokinetic Interaction Between Zidovudine and Rifampicin Following Oral Administration of the Combination at 1000 and 2200 Hours**. *Drug metabolism and drug interactions* 2001, **18**(2):123-134.

172. Kasiakou SK, Sermaides GJ, Michalopoulos A, Soteriades ES, Falagas ME: **Continuous versus intermittent intravenous administration of antibiotics: a meta-analysis of randomised controlled trials**. *The Lancet infectious diseases* 2005, **5**(9):581-589.

173. Katsube T, Wajima T, Yamano Y, Yano Y: **Pharmacokinetic/Pharmacodynamic Modeling for Concentration‐Dependent Bactericidal Activity of a Bicyclolide, Modithromycin**. *Journal of pharmaceutical sciences* 2014, **103**(4):1288-1297.

174. Kenechukwu FC, Momoh MA, Nnamani PO, Attama AA: **Solid lipid micro-dispersions (SLMs) based on PEGylated solidified reverse micellar solutions (SRMS): a novel carrier system for gentamicin**. *Drug delivery* 2015, **22**(6):710-722.

175. Kenny J, Grime K: **Pharmacokinetic consequences of time-dependent inhibition using the isolated perfused rat liver model**. *Xenobiotica* 2006, **36**(5):351-365.

176. Kenny JR, Mukadam S, Zhang C, Tay S, Collins C, Galetin A, Khojasteh SC: **Drug–drug interaction potential of marketed oncology drugs: In vitro assessment of time-dependent cytochrome P450 inhibition, reactive metabolite formation and drug–drug interaction prediction**. *Pharmaceutical research* 2012, **29**(7):1960-1976.

177. Kietzmann M, Niedorf F, Kramer S, Hoffmann M, Schneider M, Valle M, Pankow R: **Plasma and urine concentrations of marbofloxacin following single subcutaneous administration to cats**. *Berliner und Munchener tierarztliche Wochenschrift* 2010, **124**(1-2):83-88.

178. Kim DHD, Sriharsha L, Jung CW, Kamel‐Reid S, Radich JP, Lipton JH: **Comprehensive evaluation of time‐to‐response parameter as a predictor of treatment failure following imatinib therapy in chronic phase chronic myeloid leukemia: Which parameter at which time‐point does matter?** *American journal of hematology* 2010, **85**(11):856-862.

179. Kim S-J, Lee K-J, Shin Y-C, Do E, Kim S, Chun B-G, Lee M-S, Shin K-H: **Stress-induced decrease of granule cell proliferation in adult rat hippocampus: assessment of granule cell proliferation using high doses of bromodeoxyuridine before and after restraint stress**. *Molecules & Cells (Springer Science & Business Media BV)* 2005, **19**(1).

180. Kishi S, Goto N, Nakamura T, Ueda T: **Evaluation of cell-killing effects of 1-β-d-arabinofuranosylcytosine and daunorubicin by a new computer-controlled in vitro pharmacokinetic simulation system**. *Cancer research* 1999, **59**(11):2629-2634.

181. Kitamura Y, Yoshida K, Kusama M, Sugiyama Y: **A Proposal of a Pharmacokinetic/pharmacodynamic (PK/PD) Index Map for Selecting an Optimal PK/PD Index from Conventional Indices (AUC/MIC, C max/MIC, and TAM) for Antibiotics**. *Drug metabolism and pharmacokinetics* 2014, **29**(6):455-462.

182. Kloft C, Siegert W, Beyer J, Jaehde U: **Toxicity of High‐Dose Carboplatin: Ultrafiltered and Not Total Plasma Pharmacokinetics Is of Clinical Relevance**. *The Journal of Clinical Pharmacology* 2002, **42**(7):762-773.

183. Koyama I, Matsunaga T, Harada T, Kikuno A, Hokari S, Komoda T: **Ambroxol reduces LPS toxicity mediated by induction of alkaline phosphatases in rat lung**. *Clinical biochemistry* 2004, **37**(8):688-693.

184. Kroboth PD, Folan MM, Bauer KS, Tullock W, Wright CE, Sweeney JA: **Do Alprazolam‐Induced Changes in Saccadic Eye Movement and Psychomotor Function Follow the Same Time Course?** *The Journal of Clinical Pharmacology* 1998, **38**(4):337-346.

185. Kuang Y, Zhang R, Pei Q, Tan H, Guo C, Huang J, Xiang Y, Ouyang W, Duan K, Wang S: **Pharmacokinetic and pharmacodynamic study of dexmedetomidine in elderly patients during spinal anesthesia**. *International journal of clinical pharmacology and therapeutics* 2015, **53**(12):1005-1015.

186. Kurz K, Giannitsis E, Becker M, Hess G, Zdunek D, Katus HA: **Comparison of the new high sensitive cardiac troponin T with myoglobin, h-FABP and cTnT for early identification of myocardial necrosis in the acute coronary syndrome**. *Clinical Research in Cardiology* 2011, **100**(3):209-215.

187. Kuwabara T, Kobayashi S, Sugiyama Y: **Pharmacokinetics and pharmacodynamics of a recombinant human granulocyte colony-stimulating factor**. *Drug metabolism reviews* 1996, **28**(4):625-658.

188. Kuypers DR: **Immunosuppressive drug monitoring–what to use in clinical practice today to improve renal graft outcome**. *Transplant international* 2005, **18**(2):140-150.

189. Kuypers DR, Claes K, Evenepoel P, Maes B, Coosemans W, Pirenne J, Vanrenterghem Y: **Time-related clinical determinants of long-term tacrolimus pharmacokinetics in combination therapy with mycophenolic acid and corticosteroids**. *Clinical pharmacokinetics* 2004, **43**(11):741-762.

190. Kuypers DR, Claes K, Evenepoel P, Maes B, Vanrenterghem Y: **Clinical Efficacy and Toxicity Profile of Tacrolimus and Mycophenolic Acid in Relation to Combined Long‐term Pharmacokinetics in de Novo Renal Allograft Recipients**. *Clinical Pharmacology & Therapeutics* 2004, **75**(5):434-447.

191. Landoni M, Lees P: **Pharmacokinetics and pharmacodynamics of ketoprofen enantiomers in the horse**. *Journal of veterinary pharmacology and therapeutics* 1996, **19**(6):466-474.

192. Lauritzen B, Lykkesfeldt J, Friis C: **Evaluation of a single dose versus a divided dose regimen of amoxycillin in treatment of Actinobacillus pleuropneumoniae infection in pigs**. *Research in veterinary science* 2005, **79**(1):61-67.

193. Lee JH, Cho YK, Jung YS, Kim YC, Lee MG: **Time-dependent effects of Klebsiella pneumoniae endotoxin on the telithromycin pharmacokinetics in rats; restoration of the parameters in 96-hour KPLPS rats to the control levels**. *Pulmonary pharmacology & therapeutics* 2008, **21**(6):860-865.

194. Lee J-H, Na K, Song S-C, Lee J, Kuh H-J: **The distribution and retention of paclitaxel and doxorubicin in multicellular layer cultures**. *Oncology reports* 2012, **27**(4):995-1002.

195. Lehmann G, Asskali F, Boll M, Burmeister M, Marx G, Hilgers R, Förster H: **HES 130/0.42 shows less alteration of pharmacokinetics than HES 200/0.5 when dosed repeatedly†**. *British journal of anaesthesia* 2007, **98**(5):635-644.

196. Levison ME, Levison JH: **Pharmacokinetics and pharmacodynamics of antibacterial agents**. *Infectious disease clinics of North America* 2009, **23**(4):791-815.

197. Li J, Zhao J, Hamer-Maansson JE, Andersson T, Fulmer R, Illueca M, Lundborg P: **Pharmacokinetic properties of esomeprazole in adolescent patients aged 12 to 17 years with symptoms of gastroesophageal reflux disease: a randomized, open-label study**. *Clinical therapeutics* 2006, **28**(3):419-427.

198. Lilia G, Aguilera R, Cortes-Cuevas A, Rosario C, Sumano H: **Circadian serum concentrations of tylosin in broilers after feed or water medication**. *British poultry science* 2008, **49**(5):619-624.

199. Liu Y, Yoo SD, Li L, Fang L, Wen Z, Li T: **Formulation and characterization of boanmycin-loaded liposomes prepared by pH gradient experimental design**. *Drug delivery* 2012, **19**(2):90-101.

200. López‐Alvarez J, Elliott J, Pfeiffer D, Chang YM, Mattin M, Moonarmart W, Hezzell M, Boswood A: **Clinical severity score system in dogs with degenerative mitral valve disease**. *Journal of Veterinary Internal Medicine* 2015, **29**(2):575-581.

201. Lu C, Suri A, Shyu WC, Prakash S: **Assessment of cytochrome P450‐mediated drug–drug interaction potential of orteronel and exposure changes in patients with renal impairment using physiologically based pharmacokinetic modeling and simulation**. *Biopharmaceutics & drug disposition* 2014, **35**(9):543-552.

202. Lucas M, Errecalde J, Mestorino N: **Pharmacokinetics of azithromycin in lactating dairy cows with subclinical mastitis caused by Staphylococcus aureus**. *Journal of veterinary pharmacology and therapeutics* 2010, **33**(2):132-140.

203. Luo Q, Lin T, Zhang CY, Zhu T, Wang L, Ji Z, Jia B, Ge T, Peng D, Chen W: **A novel glyceryl monoolein-bearing cubosomes for gambogenic acid: preparation, cytotoxicity and intracellular uptake**. *International journal of pharmaceutics* 2015, **493**(1):30-39.

204. Lutsar I, McCracken Jr GH, Friedland IR: **Antibiotic pharmacodynamics in cerebrospinal fluid**. *Clinical Infectious Diseases* 1998:1117-1127.

205. M Aqel S, M Irshaid Y, N Gharaibeh M, A Arafat T: **The effect of ethyl acetate extract of pomelo mix on systemic exposure of verapamil in rabbits**. *Drug metabolism letters* 2011, **5**(2):92-98.

206. Mao J, Johnson TR, Shen Z, Yamazaki S: **Prediction of crizotinib-midazolam interaction using the Simcyp population-based simulator: comparison of CYP3A time-dependent inhibition between human liver microsomes versus hepatocytes**. *Drug Metabolism and Disposition* 2013, **41**(2):343-352.

207. Marinovich S, Lavorato C, Moriñigo C, Celia E, Bisignano L, Soratti M, Hansen-Krogh D: **A new prognostic index for one-year survival in incident hemodialysis patients**. *The International journal of artificial organs* 2010, **33**(10):689-699.

208. Mazzei T, Cassetta MI, Fallani S, Arrigucci S, Novelli A: **Pharmacokinetic and pharmacodynamic aspects of antimicrobial agents for the treatment of uncomplicated urinary tract infections**. *International journal of antimicrobial agents* 2006, **28**:35-41.

209. McBrien KA, Kleinman KP, Abrams AM, Prosser LA: **Use of outcomes to evaluate surveillance systems for bioterrorist attacks**. *BMC medical informatics and decision making* 2010, **10**(1):1.

210. McKellar Q, Sanchez Bruni S, Jones D: **Pharmacokinetic/pharmacodynamic relationships of antimicrobial drugs used in veterinary medicine**. *Journal of Veterinary Pharmacology and Therapeutics* 2004, **27**(6):503-514.

211. Meineke I, Gleiter CH: **Assessment of drug accumulation in the evaluation of pharmacokinetic data**. *The Journal of Clinical Pharmacology* 1998, **38**(8):680-684.

212. Menegazzi JJ, Wang HE, Lightfoot CB, Fertig KC, Chengelis NL, Sherman LD, Callaway CW: **Immediate defibrillation versus interventions first in a swine model of prolonged ventricular fibrillation**. *Resuscitation* 2003, **59**(2):261-270.

213. Michailidi C, Hayashi M, Datta S, Sen T, Zenner K, Oladeru O, Brait M, Izumchenko E, Baras A, VandenBussche C: **Involvement of epigenetics and EMT-related miRNA in arsenic-induced neoplastic transformation and their potential clinical use**. *Cancer Prevention Research* 2015, **8**(3):208-221.

214. Min DI, Chen HY, Lee MK, Ashton K, Martin MF: **Time‐Dependent Disposition of Tacrolimus and Its Effect on Endothelin‐1 in Liver Allograft Recipients**. *Pharmacotherapy: The Journal of Human Pharmacology and Drug Therapy* 1997, **17**(3):457-463.

215. Minematsu T, Lee J, Zha J, Moy S, Kowalski D, Hori K, Ishibashi K, Usui T, Kamimura H: **Time-Dependent Inhibitory Effects of (1R, 9S, 12S, 13R, 14S, 17R, 18E, 21S, 23S, 24R, 25S, 27R)-1, 14-Dihydroxy-12-(E)-2-[(1R, 3R, 4R)-4-hydroxy-3-methoxycyclohexyl]-1-methylvinyl-23, 25-dimethoxy-13, 19, 21, 27-tetramethyl-17-(2-oxopropyl)-11, 28-dioxa-4-azatricyclo [22.3. 1.04. 9] octacos-18-ene-2, 3, 10, 16-tetrone (FK1706), a Novel Nonimmunosuppressive Immunophilin Ligand, on CYP3A4/5 Activity in Humans in Vivo and in Vitro**. *Drug Metabolism and Disposition* 2010, **38**(2):249-259.

216. Mirfazaelian A, Rouini M, Dadashzadeh S: **Time dependent pharmacokinetics of albendazole in human**. *Biopharmaceutics & drug disposition* 2003, **24**(5):199-204.

217. Morgan Jr RJ, Synold TW, Gandara D, Muggia F, Scudder S, Reed E, Margolin K, Raschko J, Leong L, Shibata S: **Phase II trial of carboplatin and infusional cyclosporine in platinum-resistant recurrent ovarian cancer**. *Cancer chemotherapy and pharmacology* 2004, **54**(4):283-289.

218. Mouly S, Lown KS, Kornhauser D, Joseph JL, Fiske WD, Benedek IH, Watkins PB: **Hepatic but not intestinal CYP3A4 displays dose‐dependent induction by efavirenz in humans**. *Clinical Pharmacology & Therapeutics* 2002, **72**(1):1-9.

219. Romeu J, Balagué M, Ruiz L, Marfil S, Gatell JM, Puig T, Arnó A, Tural C, Sirera G, Clotet B: **Short-term risk for AIDS-indicator diseases predicted by plasma HIV-1 RNA and CD4+ lymphocytes**. *Scandinavian journal of infectious diseases* 1999, **31**(1):37-42.

220. Sladjana A, Gordana P, Ana S: **Emergency response time after out-of-hospital cardiac arrest**. *European journal of internal medicine* 2011, **22**(4):386-393.

221. Sosnowski M, Korzeniowska B, Tendera M: **Left ventricular mass and hypertrophy assessment by means of the QRS complex voltage-independent measurements**. *International journal of cardiology* 2006, **106**(3):382-389.

222. Tonin L, Leeb R, Del R Millán J: **Time-dependent approach for single trial classification of covert visuospatial attention**. *Journal of neural engineering* 2012, **9**(4):045011.

223. Wang F, Long G, Zhao C, Li H, Chaugai S, Wang Y, Chen C, Wang DW: **Plasma microRNA-133a is a new marker for both acute myocardial infarction and underlying coronary artery stenosis**. *Journal of translational medicine* 2013, **11**(1):1.

224. Witteveen A, Vliegen IM, Sonke GS, Klaase JM, IJzerman MJ, Siesling S: **Personalisation of breast cancer follow-up: a time-dependent prognostic nomogram for the estimation of annual risk of locoregional recurrence in early breast cancer patients**. *Breast cancer research and treatment* 2015, **152**(3):627-636.

225. Woillard J-B, Saint-Marcoux F, Monchaud C, Youdarène R, Pouche L, Marquet P: **Mycophenolic mofetil optimized pharmacokinetic modelling, and exposure-effect associations in adult heart transplant recipients**. *Pharmacological Research* 2015, **99**:308-315.

226. Xu YJ, Tappia PS, Goyal RK, Dhalla NS: **Mechanisms of the lysophosphatidic acid‐induced increase in [Ca2+] i in skeletal muscle cells**. *Journal of cellular and molecular medicine* 2008, **12**(3):942-954.

227. Uchida E, Kondo Y, Amano A, Aizawa S, Hanamura T, Aoki H, Nagamine K, Koizumi T, Maruyama N, Ishigami A: **Absorption and excretion of ascorbic acid alone and in acerola (Malpighia emarginata) juice: comparison in healthy Japanese subjects**. *Biological and Pharmaceutical Bulletin* 2011, **34**(11):1744-1747.

228. Hittmair K, Fleischmann D: **Accuracy of predicting and controlling time-dependent aortic enhancement from a test bolus injection**. *Journal of computer assisted tomography* 2001, **25**(2):287-294.

229. Witte K, Zuther P, Lemmer B: **Analysis of telemetric time series data for periodic components using DQ-FIT**. *Chronobiology international* 1997, **14**(6):561-574.

230. WATANABE J, MINAMI M, KOBAYASHI M: **Antitumor activity of TZT-1027 (soblidotin)**. *Anticancer research* 2006, **26**(3A):1973-1981.

231. Svensson US, Ashton M, Hai TN, Bertilsson L, Huong DX, Van Huong N, Niêu NT, Sy ND, Lykkesfeldt J, Công LD: **Artemisinin induces omeprazole metabolism in human beings**. *Clinical Pharmacology & Therapeutics* 1998, **64**(2):160-167.

232. Tang W, Stearns R, Wang R, Miller R, Chen Q, Ngui J, Bakshi R, Nargund R, Dean D, Baillie T: **Assessing and minimizing time-dependent inhibition of cytochrome P450 3A in drug discovery: a case study with melanocortin-4 receptor agonists**. *Xenobiotica* 2008, **38**(11):1437-1451.

233. Shahidi B, Sannes T, Laudenslager M, Maluf KS: **Cardiovascular responses to an acute psychological stressor are associated with the cortisol awakening response in individuals with chronic neck pain**. *Physiology & behavior* 2015, **150**:93-98.

234. Mustafa F, Yang T, Khan MA, Ahsan F: **Chain length‐dependent effects of alkylmaltosides on nasal absorption of enoxaparin**. *Journal of pharmaceutical sciences* 2004, **93**(3):675-683.

235. Naruhashi K, Nadai M, Nakao M, Suzuki N, Nabeshima T, Hasegawa T: **Changes in absorptive function of rat intestine injured by methotrexate**. *Clinical and Experimental Pharmacology and Physiology* 2000, **27**(12):980-986.

236. POONDRU S, DEVARAJ R, BOINPALLY RR, YAMASANI MR: **Chronopharmacokinetics of sumatriptan in healthy human subjects**. *Journal of pharmacy and pharmacology* 2000, **52**(9):1085-1090.

237. Park SI, Felipe CR, Pinheiro‐Machado PG, Garcia R, Tedesco‐Silva H, Medina‐Pestana JO: **Circadian and time‐dependent variability in tacrolimus pharmacokinetics**. *Fundamental & clinical pharmacology* 2007, **21**(2):191-197.

238. Wispelwey B: **Clinical implications of pharmacokinetics and pharmacodynamics of fluoroquinolones**. *Clinical infectious diseases* 2005, **41**(Supplement 2):S127-S135.

239. Perry TR, Schentag JJ: **Clinical Use of Ceftriaxone**. *Clinical Pharmacokinetics* 2001, **40**(9):685-694.

240. Pan T, Huang B, Zhang W, Gabos S, Huang DY, Devendran V: **Cytotoxicity assessment based on the AUC 50 using multi-concentration time-dependent cellular response curves**. *Analytica chimica acta* 2013, **764**:44-52.

241. Rietjens IM, Boersma MG, Zaleska M, Punt A: **Differences in simulated liver concentrations of toxic coumarin metabolites in rats and different human populations evaluated through physiologically based biokinetic (PBBK) modeling**. *Toxicology in Vitro* 2008, **22**(8):1890-1901.

242. Youssef AS, Parkman HP, Nagar S: **Domperidone interacts with pioglitazone but not with ondansetron via common CYP metabolism in vitro**. *Xenobiotica* 2014, **44**(9):792-803.

243. To H, Xiu DR, Hishikawa S, Uchida H, Sudoh T, Sunaga K, Sugimoto K, Higuchi S, Fujimura A, Kobayashi E: **Dosing time-dependent pharmacological effects of anti-metabolites for rat cardiac graft**. *Research communications in molecular pathology and pharmacology* 2000, **110**(5-6):319-332.

244. Rozmus J, Schultz KR, Wynne K, Kariminia A, Satyanarayana P, Krailo M, Grupp SA, Gilman AL, Goldman FD: **Early and late extensive chronic graft-versus-host disease in children is characterized by different Th1/Th2 cytokine profiles: findings of the Children's Oncology Group Study ASCT0031**. *Biology of Blood and Marrow Transplantation* 2011, **17**(12):1804-1813.

245. Agtmael MA, Gupta V, Graaf CA, Boxtel CJ: **The effect of grapefruit juice on the time‐dependent decline of artemether plasma levels in healthy subjects**. *Clinical Pharmacology & Therapeutics* 1999, **66**(4):408-414.

246. Oda K, Yamano K: **Effect of telaprevir on the metabolism and hepatic uptake of tacrolimus (FK506)**. *Biopharmaceutics & drug disposition* 2014, **35**(9):501-512.

247. Shimizu M, Uno T, Sugawara K, Tateishi T: **Effects of single and multiple doses of itraconazole on the pharmacokinetics of fexofenadine, a substrate of P‐glycoprotein**. *British journal of clinical pharmacology* 2006, **62**(3):372-376.

248. Verrier RL, Nearing BD, Ghanem RN, Olson RE, Garberich RF, Katsiyiannis WT, Gornick CC, Tang CY, Henry TD: **Elevated T‐Wave Alternans Predicts Nonsustained Ventricular Tachycardia in Association with Percutaneous Coronary Intervention in ST‐Segment Elevation Myocardial Infarction (STEMI) Patients**. *Journal of cardiovascular electrophysiology* 2013, **24**(6):658-663.

249. Nirogi R, Palacharla RC, Mohammed AR, Manoharan A, kumar Ponnamaneni R, Bhyrapuneni G: **Evaluation of metabolism dependent inhibition of CYP2B6 mediated bupropion hydroxylation in human liver microsomes by monoamine oxidase inhibitors and prediction of potential as perpetrators of drug interaction**. *Chemico-biological interactions* 2015, **230**:9-20.

250. Xing J, Kirby BJ, Whittington D, Wan Y, Goodlett DR: **Evaluation of P450 inhibition and induction by artemisinin antimalarials in human liver microsomes and primary human hepatocytes**. *Drug Metabolism and Disposition* 2012, **40**(9):1757-1764.

251. Winter H, Egizi E, Erondu N, Ginsberg A, Rouse DJ, Severynse-Stevens D, Pauli E, Everitt D: **Evaluation of pharmacokinetic interaction between PA-824 and midazolam in healthy adult subjects**. *Antimicrobial agents and chemotherapy* 2013, **57**(8):3699-3703.

252. Nakai D, Fuse E, Suzuki H, Inaba M, Sugiyama Y: **Evaluation of the efficiency of targeting of antitumor drugs: simulation analysis based on pharmacokinetic/pharmacodynamic considerations**. *Journal of drug targeting* 1996, **3**(6):443-453.

253. Mukadam S, Tay S, Tran D, Wang L, M Delarosa E, Cyrus Khojasteh S, S Halladay J, R Kenny J: **Evaluation of time-dependent cytochrome p450 inhibition in a high-throughput, automated assay: introducing a novel area under the curve shift approach**. *Drug metabolism letters* 2012, **6**(1):43-53.

254. Vieira ML, Kirby B, Ragueneau‐Majlessi I, Galetin A, Chien J, Einolf H, Fahmi O, Fischer V, Fretland A, Grime K: **Evaluation of various static in vitro–in vivo extrapolation models for risk assessment of the CYP3A inhibition potential of an investigational drug**. *Clinical Pharmacology & Therapeutics* 2014, **95**(2):189-198.

255. Thörn HA, Yasin M, Dickinson PA, Lennernäs H: **Extensive intestinal glucuronidation of raloxifene in vivo in pigs and impact for oral drug delivery**. *Xenobiotica* 2012, **42**(9):917-928.

256. Patlolla RR, Chougule M, Patel AR, Jackson T, Tata PN, Singh M: **Formulation, characterization and pulmonary deposition of nebulized celecoxib encapsulated nanostructured lipid carriers**. *Journal of Controlled Release* 2010, **144**(2):233-241.

257. Tornio A, Filppula A, Kailari O, Neuvonen M, Nyrönen T, Tapaninen T, Neuvonen P, Niemi M, Backman J: **Glucuronidation converts clopidogrel to a strong time-dependent inhibitor of CYP2C8: a phase II metabolite as a perpetrator of drug-drug interactions**. *Clinical Pharmacology & Therapeutics* 2014, **96**(4).

258. Real-Fernández F, Passalacqua I, Peroni E, Chelli M, Lolli F, Papini AM, Rovero P: **Glycopeptide-based antibody detection in multiple sclerosis by surface plasmon resonance**. *Sensors* 2012, **12**(5):5596-5607.

259. Zhuang X-M, Zhong Y-H, Xiao W-B, Li H, Lu C: **Identification and characterization of psoralen and isopsoralen as potent CYP1A2 reversible and time-dependent inhibitors in human and rat preclinical studies**. *Drug Metabolism and Disposition* 2013, **41**(11):1914-1922.

260. Smirnova MV, Vostrov SN, Strukova EV, Dovzhenko SA, Kobrin MB, Portnoy YA, Zinner SH, Firsov AA: **The impact of duration of antibiotic exposure on bacterial resistance predictions using in vitro dynamic models**. *Journal of antimicrobial chemotherapy* 2009:dkp287.

261. Xu C, Desta Z: **In vitro analysis and quantitative prediction of efavirenz inhibition of eight cytochrome P450 (CYP) enzymes: major effects on CYPs 2B6, 2C8, 2C9 and 2C19**. *Drug metabolism and pharmacokinetics* 2013, **28**(4):362-371.

262. Polasek TM, Sadagopal JS, Elliot DJ, Miners JO: **In vitro-in vivo extrapolation of zolpidem as a perpetrator of metabolic interactions involving CYP3A**. *European journal of clinical pharmacology* 2010, **66**(3):275-283.

263. Sekiguchi N, Kato M, Takada M, Watanabe H, Higashida A, Sakai S, Ishigai M, Aso Y: **In vivo approach for the evaluation of mechanism-based inhibition of cytochrome P450 3A in rats**. *Xenobiotica* 2008, **38**(4):368-381.

264. Saito Y, Nishimura Y, Kurata N, Iwase M, Aoki K, Yasuhara H: **In vivo inhibition of CYP3A-mediated midazolam metabolism by anchusan in rats**. *Journal of pharmacological sciences* 2011, **115**(3):399-407.

265. Goren Y, Davrath LR, Pinhas I, Toledo E, Akselrod S: **Individual time-dependent spectral boundaries for improved accuracy in time-frequency analysis of heart rate variability**. *IEEE transactions on biomedical engineering* 2006, **53**(1):35-42.

266. Schmid A, Petry N, Walther B, Bütikofer U, Luginbühl W, Gille D, Chollet M, McTernan PG, Gijs MA, Vionnet N: **Inflammatory and metabolic responses to high-fat meals with and without dairy products in men**. *British Journal of Nutrition* 2015, **113**(12):1853-1861.

267. Shirasaka Y, Sager JE, Lutz JD, Davis C, Isoherranen N: **Inhibition of CYP2C19 and CYP3A4 by omeprazole metabolites and their contribution to drug-drug interactions**. *Drug Metabolism and Disposition* 2013, **41**(7):1414-1424.

268. Peng JZ, Remmel RP, Sawchuk RJ: **Inhibition of murine cytochrome P4501A by tacrine: in vitro studies**. *Drug metabolism and disposition* 2004, **32**(8):805-812.

269. Tonn GR, Wong SG, Wong SC, Johnson MG, Ma J, Cho R, Floren LC, Kersey K, Berry K, Marcus AP: **An inhibitory metabolite leads to dose-and time-dependent pharmacokinetics of (R)-N-{1-[3-(4-ethoxy-phenyl)-4-oxo-3, 4-dihydro-pyrido [2, 3-d] pyrimidin-2-yl]-ethyl}-N-pyridin-3-yl-methyl-2-(4-trifluoromethoxy-phenyl)-acetamide (AMG 487) in human subjects after multiple dosing**. *Drug Metabolism and Disposition* 2009, **37**(3):502-513.

270. Stroh M, Talaty J, Sandhu P, McCrea J, Patnaik A, Tolcher A, Palcza J, Orford K, Breidinger S, Narasimhan N: **Lack of meaningful effect of ridaforolimus on the pharmacokinetics of midazolam in cancer patients: Model prediction and clinical confirmation**. *The Journal of Clinical Pharmacology* 2014, **54**(11):1256-1262.

271. Satoh S, Kagaya H, Saito M, Inoue T, Miura M, Inoue K, Numakura K, Tsuchiya N, Tada H, Suzuki T: **Lack of tacrolimus circadian pharmacokinetics and CYP3A5 pharmacogenetics in the early and maintenance stages in Japanese renal transplant recipients**. *British journal of clinical pharmacology* 2008, **66**(2):207-214.

272. Park HD, Kim HJ, Oh YS, Kim IC, Zu Kim Y, Koh HC, Shin IC, Lee YH, Lee CH: **LB30057, an orally effective direct thrombin inhibitor, prevents arterial and venous thrombosis in Rats and Dogs**. *Archives of pharmacal research* 2003, **26**(3):224-231.

273. Pea F, Viale P, Lugano M, Pavan F, Scudeller L, Della Rocca G, Furlanut M: **Linezolid disposition after standard dosages in critically ill patients undergoing continuous venovenous hemofiltration: a report of 2 cases**. *American journal of kidney diseases* 2004, **44**(6):1097-1102.

274. Nikisch G, Mathé AA, Czernik A, Thiele J, Bohner J, Eap CB, Ågren H, Baumann P: **Long-term citalopram administration reduces responsiveness of HPA axis in patients with major depression: relationship with S-citalopram concentrations in plasma and cerebrospinal fluid (CSF) and clinical response**. *Psychopharmacology* 2005, **181**(4):751-760.

275. Skjøt-Arkil H, Clausen RE, Nguyen QHT, Wang Y, Zheng Q, Martinez FJ, Hogaboam CM, Han M, Klickstein LB, Larsen MR: **Measurement of MMP-9 and-12 degraded elastin (ELM) provides unique information on lung tissue degradation**. *BMC pulmonary medicine* 2012, **12**(1):1.

276. Ung D, Parkman H, Nagar S: **Metabolic interactions between prokinetic agents domperidone and erythromycin: an in vitro analysis**. *Xenobiotica* 2009, **39**(10):749-756.

277. Rittenhouse KD, Peiffer Jr RL, Pollack GM: **Microdialysis evaluation of the ocular pharmacokinetics of propranolol in the conscious rabbit**. *Pharmaceutical research* 1999, **16**(5):736-742.

278. Rakic Ignjatovic A, Miljkovic B, Todorovic D, Timotijevic I, Pokrajac M: **Moclobemide monotherapy vs. combined therapy with valproic acid or carbamazepine in depressive patients: a pharmacokinetic interaction study**. *British journal of clinical pharmacology* 2009, **67**(2):199-208.

279. Takahashi Y, Yamaoka K, Nishikawa M, Takakura Y: **Moment analysis for kinetics of gene silencing by RNA interference**. *Biotechnology and bioengineering* 2006, **93**(4):816-819.

280. van Warmerdam LJ, van Tellingen O, Wim W, Rodenhuis S, Maes RA, Beijnen JH: **Monitoring Carboplatin Concentrations in Saliva: A Replacement for Plasma Ultrafiltrate Measurements?** *Therapeutic drug monitoring* 1995, **17**(5):465-470.

281. Shandil RK, Jayaram R, Kaur P, Gaonkar S, Suresh B, Mahesh B, Jayashree R, Nandi V, Bharath S, Balasubramanian V: **Moxifloxacin, ofloxacin, sparfloxacin, and ciprofloxacin against Mycobacterium tuberculosis: evaluation of in vitro and pharmacodynamic indices that best predict in vivo efficacy**. *Antimicrobial agents and chemotherapy* 2007, **51**(2):576-582.

282. Xing J, Bai K, Liu T, Wang R, Zhang L, Zhang S: **The multiple-dosing pharmacokinetics of artemether, artesunate, and their metabolite dihydroartemisinin in rats**. *Xenobiotica* 2011, **41**(3):252-258.

283. Zhanel GG, Calic D, Schweizer F, Zelenitsky S, Adam H, Lagacé-Wiens PRS, Rubinstein E, Gin AS, Hoban DJ, Karlowsky JA: **New Lipoglycopeptides**. *Drugs* 2010, **70**(7):859-886.

284. Tam VH, Nikolaou M: **A novel approach to pharmacodynamic assessment of antimicrobial agents: new insights to dosing regimen design**. *PLoS Comput Biol* 2011, **7**(1):e1001043.

285. Tomkinson HK, Kemp JV, Wollseifen T, Morris T, Oliver SD: **An open-label, randomized, single-center, two-period, phase I, crossover study of the effect of zibotentan (ZD4054) on the pharmacokinetics of midazolam in healthy male volunteers**. *Clinical therapeutics* 2010, **32**(7):1372-1386.

286. Nicolau DP: **Optimizing outcomes with antimicrobial therapy through pharmacodynamic profiling**. *Journal of infection and chemotherapy* 2003, **9**(4):292-296.

287. Oellerich M, Shipkova M, Schütz E, Wieland E, Weber L, Tönshoff B, Armstrong VW, Recipients GSGoMMTiPRT: **Pharmacokinetic and metabolic investigations of mycophenolic acid in pediatric patients after renal transplantation: implications for therapeutic drug monitoring**. *Therapeutic drug monitoring* 2000, **22**(1):20-26.

288. Tiseo P, Rogers S, Friedhoff L: **Pharmacokinetic and pharmacodynamic profile of donepezil HCl following evening administration**. *British journal of clinical pharmacology* 1998, **46**:13-18.

289. Piscitelli SC, Forrest A, Vogel S, Chaitt D, Metcalf J, Stevens R, Baseler M, Davey RT, Kovacs JA: **Pharmacokinetic modeling of recombinant interleukin‐2 in patients with human immunodeficiency virus infection**. *Clinical Pharmacology & Therapeutics* 1998, **64**(5):492-498.

290. Tsuchiya H, Kuwata K, Nagayama S, Yamashita K, Kamiya H, Harashima H: **Pharmacokinetic modeling of species-dependent enhanced bioavailability of trifluorothymidine by thymidine phosphorylase inhibitor**. *Drug metabolism and pharmacokinetics* 2004, **19**(3):206-215.

291. Theuretzbacher U, Ihle F, Derendorf H: **Pharmacokinetic/pharmacodynamic profile of voriconazole**. *Clinical pharmacokinetics* 2006, **45**(7):649-663.

292. Toutain P-L, Del Castillo JR, Bousquet-Mélou A: **The pharmacokinetic–pharmacodynamic approach to a rational dosage regimen for antibiotics**. *Research in veterinary science* 2002, **73**(2):105-114.

293. Turnheim K, Krivanek P, Oberbauer R: **Pharmacokinetics and pharmacodynamics of allopurinol in elderly and young subjects**. *British journal of clinical pharmacology* 1999, **48**(4):501.

294. Waldman SA, Vitow C, Osborne B, Gillen L, Argentieri DC, Wong FA, Smith IL, Chow AT, Misiti J, Bjornsson TD: **Pharmacokinetics and pharmacodynamics of tepoxalin after single oral dose administration to healthy volunteers**. *The Journal of Clinical Pharmacology* 1996, **36**(5):462-468.

295. Zozaya H, Gutierrez L, Bernad MJ, Sumano H: **Pharmacokinetics of a peroral single dose of two long-acting formulations and an aqueous formulation of doxycycline hyclate in horses**. *Acta Veterinaria Scandinavica* 2013, **55**(1):1.

296. Shih P-S, Huang J-D: **Pharmacokinetics of midazolam and 1′-hydroxymidazolam in Chinese with different CYP3A5 genotypes**. *Drug metabolism and disposition* 2002, **30**(12):1491-1496.

297. Sturmer A, Mehta N, Giacchi J, Cagatay T, Tavakkol R, Mitta S, Fitzpatrick L, Wald J, Trang J, Stern W: **Pharmacokinetics of Oral Recombinant Human Parathyroid Hormone [rhPTH (1–31) NH2] in Postmenopausal Women with Osteoporosis**. *Clinical pharmacokinetics* 2013, **52**(11):995-1004.

298. Saadeddin A, Torres-Molina F, Cárcel-Trullols J, Araico A, Peris J-E: **Pharmacokinetics of the time-dependent elimination of all-trans-retinoic acid in rats**. *AAPS PharmSci* 2004, **6**(1):1-9.

299. Schmitt‐Hoffmann A, Roos B, Sauer J, Spickermann J, Stoeckel K, Edwards D, van de Wetering J, Coenraads P, Maares J: **Pharmacokinetics, efficacy and safety of alitretinoin in moderate or severe chronic hand eczema**. *Clinical and experimental dermatology* 2011, **36**(s2):29-34.

300. Yamamoto K, Noda K, Yoshimura A, Fukuoka M, Furuse K, Niitani H: **Phase I study of E7010**. *Cancer chemotherapy and pharmacology* 1998, **42**(2):127-134.

301. Yeo KR, Jamei M, Yang J, Tucker GT, Rostami-Hodjegan A: **Physiologically based mechanistic modelling to predict complex drug–drug interactions involving simultaneous competitive and time-dependent enzyme inhibition by parent compound and its metabolite in both liver and gut—the effect of diltiazem on the time-course of exposure to triazolam**. *European Journal of Pharmaceutical Sciences* 2010, **39**(5):298-309.

302. Xia B, Barve A, Heimbach T, Zhang T, Gu H, Wang L, Einolf H, Alexander N, Hanna I, Ke J: **Physiologically based pharmacokinetic modeling for assessing the clinical drug–drug interaction of alisporivir**. *European Journal of Pharmaceutical Sciences* 2014, **63**:103-112.

303. Skolnick P, Krieter P, Tizzano J, Basile A, Popik P, Czobor P, Lippa A: **Preclinical and clinical pharmacology of DOV 216,303, a “triple” reuptake inhibitor**. *CNS drug reviews* 2006, **12**(2):123-134.

304. Nicolau D: **Predicting antibacterial response from pharmacodynamic and pharmacokinetic profiles**. *Infection* 2001, **29**:11-15.

305. Vieira ML, Zhao P, Berglund E, Reynolds K, Zhang L, Lesko L, Huang SM: **Predicting Drug Interaction Potential With a Physiologically Based Pharmacokinetic Model: A Case Study of Telithromycin, a Time‐Dependent CYP3A Inhibitor**. *Clinical Pharmacology & Therapeutics* 2012, **91**(4):700-708.

306. Wu F, Gaohua L, Zhao P, Jamei M, Huang S-M, Bashaw ED, Lee S-C: **Predicting nonlinear pharmacokinetics of omeprazole enantiomers and racemic drug using physiologically based pharmacokinetic modeling and simulation: application to predict drug/genetic interactions**. *Pharmaceutical research* 2014, **31**(8):1919-1929.

307. Yeo KR, Walsky R, Jamei M, Rostami-Hodjegan A, Tucker G: **Prediction of time-dependent CYP3A4 drug–drug interactions by physiologically based pharmacokinetic modelling: impact of inactivation parameters and enzyme turnover**. *European Journal of Pharmaceutical Sciences* 2011, **43**(3):160-173.

308. Alcan T, Ceylanoğlu C, Baysal B: **The relationship between digital model accuracy and time-dependent deformation of alginate impressions**. *The Angle Orthodontist* 2009, **79**(1):30-36.

309. Slavik RS, Jewesson PJ: **Selecting antibacterials for outpatient parenteral antimicrobial therapy**. *Clinical pharmacokinetics* 2003, **42**(9):793-817.

310. Solovyova AS, Nöllmann M, Mitchell TJ, Byron O: **The solution structure and oligomerization behavior of two bacterial toxins: pneumolysin and perfringolysin O**. *Biophysical journal* 2004, **87**(1):540-552.

311. Thummel KE, Lin YS: **Sources of Interindividual Variability**. In: *Enzyme Kinetics in Drug Metabolism: Fundamentals and Applications.* edn. Edited by Nagar S, Argikar AU, Tweedie JD. Totowa, NJ: Humana Press; 2014: 363-415.

312. Xu H, Loboz KK, Gross AS, McLachlan AJ: **Stereoselective analysis of hydroxybupropion and application to drug interaction studies**. *Chirality* 2007, **19**(3):163-170.

313. Park SI, Felipe CR, Pinheiro‐Machado PG, Garcia R, Fernandes FB, Casarini DE, Tedesco‐Silva H, Medina‐Pestana JO: **Tacrolimus pharmacokinetic drug interactions: effect of prednisone, mycophenolic acid or sirolimus**. *Fundamental & clinical pharmacology* 2009, **23**(1):137-145.

314. Marović D, Matić S, Kelić K, Klarić E, Rakić M, Tarle Z: **Time dependent accuracy of dental radiometers**. *Acta Clinica Croatica* 2013, **52**(2.):173-180.

315. Murakami T, Kusachi S, Murakami M, Sano I, Uesugi T, Murakami M, Hirami R, Kajiyama A, Kondo J, Tsuji T: **Time-dependent changes of serum carboxy-terminal peptide of type I procollagen and carboxy-terminal telopeptide of type I collagen concentrations in patients with acute myocardial infarction after successful reperfusion: correlation with left ventricular volume indices**. *Clinical chemistry* 1998, **44**(12):2453-2461.

316. Rolf P, Bourbeau M, Campbell P, Seguin I, Chauhan BM, Foster BC, Cameron DW: **Time‐Dependent Interaction Between Lopinavir/Ritonavir and Fexofenadine**. *The Journal of Clinical Pharmacology* 2006, **46**(7):758-767.

317. Onyeji CO, Ogunbona FA: **Time-dependent variability of chloroquine secretion into human saliva**. *Pharmacy World and Science* 1996, **18**(6):211-216.

318. Zientek M, Dalvie D: **Use of a multistaged time-dependent inhibition assay to assess the impact of intestinal metabolism on drug-drug interaction potential**. *Drug Metabolism and Disposition* 2012, **40**(3):467-473.

319. Salvan A, Thomaseth K, Bortot P, Sartori N: **Use of a toxicokinetic model in the analysis of cancer mortality in relation to the estimated absorbed dose of dioxin (2, 3, 7, 8-tetrachlorodibenzo-p-dioxin, TCDD)**. *Science of the Total Environment* 2001, **274**(1):21-35.

320. Uesawa Y, Mohri K: **The use of heat treatment to eliminate drug interactions due to grapefruit juice**. *Biological and Pharmaceutical Bulletin* 2006, **29**(11):2274-2278.

321. Shirasaki Y, Ito Y, Kikuchi M, Imamura Y, Hayashi T: **Validation studies on blood collection from the jugular vein of conscious mice**. *Journal of the American Association for Laboratory Animal Science* 2012, **51**(3):345-351.

322. Díaz I, Hubbard A, Decker A, Cohen M: **Variable importance and prediction methods for longitudinal problems with missing variables**. *PloS one* 2015, **10**(3):e0120031.

323. Zimmerman H, Thebault J, Duvauchelle T, Mignot A, Renoux A, Gualano V: **Pharmacokinetics of Estradiol Valerate 2mg+ Dienogest 2mg (Climodien® 2/2) after Single and Repeated Oral Administration in Healthy Postmenopausal Women**. *Clinical drug investigation* 2000, **20**(2):123-134.
